# Exposure to Environmental Chemicals and Infertility Among US Reproductive-Aged Women

**DOI:** 10.3390/ijerph21121541

**Published:** 2024-11-21

**Authors:** Valerie Martinez, Irene H. Yen, Camila Alvarez, Andrew D. Williams, Sandie Ha

**Affiliations:** 1 Public Health Department, School of Social Sciences, Humanities and Arts, Health Science Research Institute, University of California, Merced, CA 95343, USA; vmartinez44@ucmerced.edu (V.M.); iyen@ucmerced.edu (I.H.Y.); 2 Department of Sociology, University of California, San Diego, CA 92122, USA; calvarez10@ucsd.edu; 3 Public Health Program, School of Medicine and Health Sciences, University of North Dakota, Grand Forks, ND 58202, USA; andrew.d.williams@und.edu

**Keywords:** environmental pollutants, cadmium, hexachlorobenzene, oxychlordane, PBB-153, female fertility, reproductive health

## Abstract

Environmental chemical exposure has been rising over the past few decades but its impact on fertility remains uncertain. We assessed exposures to 23 common chemicals across a range of sociodemographic characteristics and their relationship with self-reported infertility. The analytic sample was non-pregnant women aged 18–49 years without a history of hysterectomy or oophorectomy (n = 2579) from the National Health and Nutrition Examination Survey (2013–2016). Environmental chemical exposure was assessed with biospecimens and dichotomized as high and low levels of exposure based on the median. Logistic regression models estimated the adjusted odds ratio (aOR) and 95% confidence intervals (CIs) for the association between high levels of exposure and infertility, adjusted for age, race, education level, family income, and smoking status. We observed associations between infertility and cadmium [aOR: 1.88; 95% CI: 1.02–3.47] and arsenic [aOR: 1.88 (1.05–3.36)]. Two pesticides hexachlorobenzene [OR: 2.04 (1.05–3.98)] and oxychlordane [OR: 2.04 (1.12–3.69)] were also associated with infertility in unadjusted analyses. There were negative associations with two Per- and polyfluoroalkyl substances with n-perfluorooctanoic acid [aOR: 0.51: (0.30–0.86)] and n-perfluorooctane sulfonic acid [aOR: 0.51: (0.26–0.97). Specific chemicals may contribute to infertility risk, highlighting the need for targeted public health strategies to mitigate exposure.

## 1. Introduction

Infertility is defined as the inability to achieve pregnancy after 12 months of regular unprotected sex for women ≤35 years of age and six months for women >35 years [1]. Approximately 19% of heterosexual women aged 15–49 with no prior births are unable to become pregnant after one year of trying in the United States (US) [1]. The rise in infertility is partially reflected in the increased rates of assisted reproductive technology use, which rose from 0.7% in 1998 to 2.3% in 2021 in the US [2,3]. Infertility has increased risk for adverse health implications beyond a timely pregnancy, such as lower Apgar scores, low umbilical vein pH, and neonatal intensive care requirements [4]. Infertility often results in significant psychological and social burdens, including experiences of divorce, and enduring social stigma that can lead to isolation and psychological distress [5]. Additionally, infertility treatments can be financially burdensome, with median per-person costs ranging from USD 1182 for medications alone to USD 24,373 for an infertility treatment service such as in vitro fertilization [6]. Common individual risk factors for female infertility include genetic disorders, chromosomal abnormalities, ovulatory disorders, tubal factors, endometriosis, lifestyle choices, and advanced age [7,8,9,10,11,12]. Low fertility rates can also be attributed to factors such as delayed childbearing and smaller ideal family sizes [13,14]. It is also useful to distinguish between fertility, the actual production of offspring, and fecundity, a primary determinant of fertility, which is defined as the biological capacity to conceive or sustain a pregnancy to live birth [15]. Environmental exposures may contribute to infertility primarily by impairing fecundity through mechanisms like hormonal disruption, potentially reducing the body’s reproductive capacity [16,17,18]. By impacting fecundity, environmental exposures indirectly affect fertility outcomes, as limitations in biological capacity can ultimately influence the likelihood of achieving a successful pregnancy. Understanding this distinction allows us to explore how environmental exposures might impact reproductive health both by limiting the body’s reproductive potential and by influencing fertility-related outcomes.

While the link between the aforementioned risk factors and infertility are well-documented, up to one third of infertility cases are unexplained [19]. A growing body of evidence suggests that environmental chemical exposure may be a key factor in infertility [20,21,22,23,24]. Environmental chemicals refer to chemical compounds or elements that are present in the air, water, food, soil, dust, or any other environmental medium, such as consumer products [25]. The US, the second largest chemical producer, accounting for 13% of global production, has registered over 80,000 chemicals through the National Toxicology Program, with 2000 new chemicals introduced annually—most of which are not tested for health effects [26,27]. Despite many being banned or restricted elsewhere due to reproductive or carcinogenic toxicity, many remain in production and use in the US [28,29]. Meanwhile, increased industrial activities have increased exposure to toxic-heavy metals such as cadmium and arsenic [30,31,32]. These chemicals may increase oxidative stress, systemic inflammation, and alter hormonal activities in females [33]. Previous studies investigating the link between environmental chemicals and fertility outcomes have reported inconsistent findings with both positive and null associations [17,34,35,36]. This inconsistency necessitates more research to further investigate the association between environmental chemicals and infertility.

Meanwhile, sociodemographic disparities in environmental chemical exposures are evident [37]. These differences may be linked to cultural practices and/or retail redlining, which selectively serves or offers differential services/goods based on demographics, where access to safer alternatives is limited among people of color and low-income communities [38,39,40,41,42]. In a study of women in New York City, African American and African Caribbean women had the highest usage of hair products, and as indicated by ingredient labels, were more likely to include endocrine-disrupting chemicals [43]. A California study found that Latina women used makeup most frequently, averaging more days per week compared to women of other races/ethnicities, potentially leading to elevated levels of parabens in their blood [44]. Despite the increased population exposures and the potential exposure disparities across demographic characteristics, our understanding of this pattern is limited [45]. Furthermore, given the increasing diversity of our population and the complexity of environmental threats, it is critical to identify potentially high-risk population(s). Such knowledge allows for a more comprehensive understanding of how these chemicals impact different groups and informs more inclusive and effective prevention strategies.

In this US representative cross-sectional study, we characterized environmental chemical exposures in US reproductive-aged women and explored whether exposures vary by sociodemographic factors. Additionally, we assessed the relationship between exposure to environmental chemicals and self-reported infertility. We hypothesize that exposures to environmental chemicals vary by sociodemographic characteristics and are positively associated with the odds of self-reported infertility in US reproductive-aged women.

## 2. Materials and Methods

### 2.1. Data and Participants

**Figure 1:**
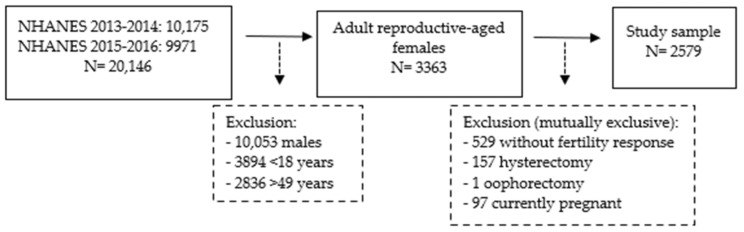
Study sample selection, NHANES (2013–2016).

Conducted by the Centers for Disease Control and Prevention and the National Center for Health Statistics, the National Health and Nutrition Examination Survey (NHANES) is a cross-sectional, nationally representative survey designed to assess the health and nutritional status of adults and children in the US [46]. NHANES utilizes a complex, multistage, probability sampling design to select participants representative of the civilian, non-institutionalized US population. The study collects comprehensive data from self-reports; medical, dental, and physiological assessments; as well as laboratory tests. For this study, we utilized cycles 2013–2014 and 2015–2016 (N = 20,146) as they represent the most recent data containing the chemicals of interest. After excluding males (n = 10,053); children below 18 (n = 3894) and adults over 49 years of age (n = 2836); women who were pregnant at the time of survey (n = 97) or had a history of hysterectomy (n = 157) or oophorectomy (n = 1); and those who did not answer the infertility question (n = 529), the final analytic sample includes 2579 women (Figure 1). Although the female reproductive age spans from 15 to 49 years according to the World Health Organization, the reproductive health questionnaire, which includes infertility information, was only administered to women over 18 [47]. The study was exempted by our Institutional Review Board.

### 2.2. Exposure Assessment

The primary exposures of interest were 23 ubiquitous environmental chemicals belonging to 6 chemical classes assessed in the NHANES (Table S1). These chemical classes—including brominated flame retardants, volatile organic compounds, cotinine (a biomarker of tobacco smoke exposure), metals, pesticides, bisphenol A, and per- and polyfluoroalkyl substances—were selected due to their widespread environmental presence and established associations with adverse health outcomes, including endocrine disruption, reproductive toxicity, and impacts on fertility [34,48,49,50,51]. Despite how ubiquitous most of these chemicals are, they remain understudied. For example, studies have shown how brominated flame retardants (BFRs) are frequently detected in the US general population; however, few studies on the human health effects of BFRs on infertility exist [52,53,54]. Most chemicals were measured in randomly selected subsets of the overall sample, which included about one-third of our sample [55]. The chemicals were assessed using urinary, blood, or serum samples (Table S1). Details regarding the methods of data collection for these chemicals have been previously published [55]. Briefly, blood plasma samples were vortexed, diluted, and then were measured by inductively coupled plasma mass spectrometry. Urinary samples (24 h) were analyzed using on-line solid-phase extraction coupled with high-performance liquid chromatography and tandem mass spectrometry. Blood serum samples were collected in non-anticoagulant-containing (red top) vacuum tubes and prepared by a standard protocol. Regardless of specimen type, concentrations of chemicals were available as continuous variables. Due to the non-normal distribution and the low prevalence of infertility, we created a dichotomized variable (high/low) for each chemical based upon the median, where high and low levels of exposure were defined as above and at or below the chemical specific sample distribution, respectively.

Since the exposure definition cut-off at the median is arbitrary, we also considered a different cut-off at the 75th percentile. Additionally, we constructed an exposure score, where a score of 1 was assigned to each participant for each chemical they were considered ‘highly exposed’ [e.g., >50th (or 75th) percentile]. Each participant can have a score ranging from 0, where they were not highly exposed to any of the 23 chemicals, to 23, where they were highly exposed to all 23 chemicals. This score was then analyzed as an independent variable both continuously and in categories defined by quartiles for both the 50th and 75th percentile.

### 2.3. Outcome Assessment

The primary outcome was self-reported infertility and was assessed using the question “Have you ever attempted to become pregnant over a period of at least a year without becoming pregnant?” If a participant responded “Yes”, then they were categorized as “infertile”; if a participant responded “No”, then they were categorized as “fertile.” The questionnaire was conducted via computer-assisted personal interviews administered by qualified interviewers in the participant’s residence. Participants who did not speak English or Spanish had interpreters [56].

### 2.4. Sociodemographic and Other Characteristics

For sociodemographic characteristics, we included age (in years): 18–29, 30–39, 40–49; race/ethnicity: Non-Hispanic (NH) White, NH Black, Hispanic, NH Asian, Other/Multiple race; educational levels: less than high school, high school graduates, some college or associates in arts (AA) degree, college graduate or more; annual family income: <USD 45k, USD 45–USD 99k, ≥USD 100k. Other factors included health status: very good/excellent, good, and fair/poor; body mass index (BMI): underweight, normal, overweight, obese; smoking status: never smoker, former smoker, current smoker; had at least 12 alcohol drinks in any one year: yes, no. These factors were all self-reported, except for BMI, which was measured through physical examinations at the mobile examination center. We identified confounders through a directed acyclic graph (DAG) (Figure S1).

### 2.5. Statistical Analysis

We combined data from two cycles using appropriate sampling weights determined by the NHANES criteria and documentation [40]. In the chemical analyses, we used additional weights that accounted for the subset of participants with chemical data. Kruskal–Wallis tests compared the difference in chemical exposures across the different sociodemographic variables. We used logistic regression models to estimate the odds ratios (OR) and 95% confidence intervals (CIs) for the associations between specific environmental chemicals and self-reported infertility, comparing those in the high-exposed group to those in the low-exposed group. We fit a series of regression models. Model 1 was an unadjusted model that only accounts for complex probability weighting. Model 2 adjusted for complex sampling and potential confounders identified by our DAG. Model 3 included covariates that were associated with both the exposures and the outcome of interest based on exploratory analyses. Alpha was set at 0.05 for statistical significance. We tested interaction terms between each chemical and age, race, education, and family income to identify susceptible groups, but did not detect any meaningful effect modification and only reported main effects. Statistical analyses were conducted using SAS Version 9.2 (Cary, NC, USA).

## 3. Results

The estimated prevalence of self-reported infertility in the study population was 12.6% (95% CI: 11.0–14.2), which is similar to the rate of 12.5% that was reported in other NHANES data for the same period [57]. The majority of study participants were 18–29 years old, NH White, married, had at least some college education, had an annual family income < USD 45,000, had at least 12 alcoholic drinks in any one year, reported having an excellent/very good health status, were never smokers, and had a BMI < 25 (Table 1). Compared to their counterparts, self-reported infertility was significantly more prevalent among women who were aged 40 to 49 years (19.7% vs. 5.6% in women 18–29), had an income of over USD 100,000 (16.6% vs. 9.9% among those who had <45K), who were married or cohabiting (16.9% vs. 7.6% among single/divorced/widowed women), or were obese (17.8% vs. 10% among those with normal weight) (Table 1).

Tables S2–S11 describe the distribution of environmental chemical exposures by sociodemographic characteristics including age, race/ethnicity, education, family household income, marital status, general health status, BMI, smoking status, alcohol use, and infertility status, respectively. In general, we did not observe consistent patterns of differential exposure across sociodemographic characteristics for many chemicals except in a few instances. Particularly, median concentrations of many brominated flame retardants; metals including cadmium lead, and mercury; and PFAs were generally higher among women who were older (Table S2). Cotinine levels were higher among women who were younger, NH Black, had less education or income, were not married/cohabiting, had more extreme BMIs, reported fair/poor health, were a current smoker, or alcohol consumer. Metal concentrations were higher among women who were older, a former/current smoker, alcohol consumer, and those who reported infertility. BPA levels were higher among women who were younger, NH Black, had less income, were not married/cohabiting, had a worse health status, were obese, a current smoker, or an alcohol consumer. Furthermore, pesticide exposures were also generally higher among women who were older, a former/current smoker, alcohol consumer, and those who reported infertility.

Table 2 presents the association between environmental chemicals and the odds of self-reported infertility. Model 1 (unadjusted) indicated that women who had high levels of exposure to PBB-153, a common BFR, had 2.09 times the odds of reported infertility [OR:2.09, 95% CI: 1.24–3.53] compared with low-exposed women. These associations were not statistically significant after adjusting for covariates (aOR_Model2_: 1.12; 95% CI: 0.54–2.33, aOR_Model3_: 1.44; 95% CI: 0.73–2.83). In addition, women with high levels of exposure to the metal cadmium had 1.88 times the odds of self-reported infertility [aOR_Model2_: 1.88, 95% CI: 1.02–3.47] compared with those with low levels of exposure.

Meanwhile, women with high levels of exposure to pesticides hexachlorobenzene [OR_Model1_: 2.04, 95% CI: 1.05–3.98] and oxychlordane [OR_Model1_: 2.04, 95% CI: 1.12–3.69] had about twice the odds of reporting infertility compared with those with low levels of exposure. In adjusted models, these associations were not statistically significant, as demonstrated by (aOR_Model2_: 1.58, 95% CI: 0.55–4.50; and aOR_Model3_: 1.48, 95% CI: 0.52–4.19) and (aOR_Model2_: 1.09, 95% CI: 0.49–2.41; and aOR_Model3_: 1.29, 95% CI: 0.63–3.07), respectively, for hexachlorobenzene and oxychlordane.

We also observed inverse associations with two per- and polyfluoroalkyl substances (PFAs). Women with high levels of exposure to n-perfluorooctanoic acid (n-PFOA) had 0.46 times the odds of reporting infertility [aOR_Model2_: 0.46, 95% CI: 0.26–0.81] compared to women with low levels of exposure. Similar results were found for n-perfluorooctane sulfonic acid (n-PFOS) [aOR_Model2_: 0.46, 95% CI: 0.24–0.88).

The results were generally consistent when the 75th percentile was used as the cut-off (Table 3). After adjusting for confounders, arsenic was positively associated with infertility [aOR_Model2_: 1.88, 95% CI: 1.05–3.36]. The inverse associations with PFAs mostly disappeared except for the associations with n-PFOS in Model 2 in Table 3 [aOR: 0.51, 95% CI: 0.28–0.92]. Meanwhile, analysis of the exposure scores suggested that those with higher scores had higher odds of self-reported infertility, although none of the estimates were statistically significant, potentially due to the moderate prevalence of infertility (Table S12).

## 4. Discussion

This study characterized sociodemographic variations in chemical exposures and their association with self-reported infertility. In terms of sociodemographic associations, self-reported infertility was more prevalent among older women, those with higher incomes, married women, and those with higher BMI. These variations in risk may reflect underlying physiological mechanisms affecting fertility, such as age-related declines in oocyte quantity and quality, hormonal imbalances associated with obesity, and the tendency for individuals with higher education and income to delay childbearing, which is linked to age-related reductions in fertility [58,59]. While we observed no consistent patterns in exposures across most sociodemographic characteristics, data suggested specific instances of potential variation. Chemical burdens were generally higher among older women, those who were non-White, and those with lower sociodemographic indicators, but in some cases, findings were mixed. We also observed positive associations between self-reported infertility and some BFRs, heavy metals, and pesticides.

Polybrominated diphenyl ether (PBDE) flame retardants and metals such as cadmium and lead were higher in older reproductive-aged women compared with their younger counterparts. Another NHANES analysis shows that adults aged ≥ 60 had twice the likelihood of having serum PBDE-47 levels > 95th percentile compared with those aged 20–59 [52]. Other studies observed cadmium in higher concentrations among older reproductive-aged women [60,61]. These trends may suggest age-related differences in the exposure level, absorption, metabolism, and/or excretion of PBDEs and metal, as well as their ability to accumulate [62]. PBDEs are used as flame retardants and are commonly found in consumer products like electronics, textiles, and upholstered furniture. Although certain US manufacturers have voluntarily stopped producing some PBDEs, these chemicals persist, and, with exposure, can accumulate in human adipose tissue [63,64]. For cadmium, age-related slowing of elimination rate, coupled with the long half-life (up to 38 years in the kidneys), can cause more accumulation in older women [65]. Because BFRs and metals have been linked to many health outcomes across the lifespan, including thyroid disorders, diabetes, cardiovascular disease, and cancers, higher concentrations may contribute to a higher health burden in older women [66,67,68].

Higher pesticide concentrations were observed among NH Black and NH Asian women, and elevated BFR levels among women of other and multi-race backgrounds. These disparities can be explained by structural racism and classism identified as a system shaped by historical, institutional, cultural, or behavioral societal behaviors that consistently harm and oppress communities of color, which has resulted in notable disparities in exposure to various environmental pollutants [69]. Studies suggested that communities of color are more exposed to pesticides and other pollutants, potentially due to residing near agricultural areas and indoor use of pesticides [69,70]. Similarly, Sjodin et al. noted lower PBDE-47 and PBDE-99 in White populations compared to Mexican American and Black populations [52]. Differences in PBDE exposure can be attributed to variations in housing quality, furniture, and dietary habits, particularly the consumption of contaminated animal fats [71,72].

Meanwhile, BPA levels were higher among women who were younger or had a lower income, were NH Black, obese, were single/divorced/widow, or reported worse health status. BPA, commonly found in shatterproof windows, eyewear, water bottles, epoxy resins that cover metal cans, and many other sources, was detected in about 89% of US women aged 16 to 49 in 2011–2012 [73]. Previous research has found that BPA levels have been higher among Black females [74,75,76] and lower income groups [77], potentially due to limited access to fresh food, leading to an increased reliance on processed food, which is typically packaged in plastics or cans with elevated BPA levels [78].

In addition to exposure disparities, the positive associations between self-reported infertility and BFRs, metals, and pesticides are concerning. BFRs are added to products like foam, textiles, and plastics to prevent fires but are not chemically bound to these materials [79]. They can enter the environment through volatilization, leaching, or product degradation. PBDEs are long-lasting and can be found in aquatic sediments, house dust, and various animals, especially fish, where they accumulate [79]. Our findings are consistent with other studies that observed associations between BFR exposure and adverse reproductive outcome exposure, such as longer time to pregnancy [80,81]. Meanwhile, a study examined in utero exposure to PBB-153 from an industrial accident in 1973 Michigan and infertility and observed no association [82]. This lack of observed association could possibly be attributed to the young age of participants (23.3 years), which might not capture the full scope of infertility as fertility issues often become more apparent with increasing age. Also, the analysis of Michigan data was limited to married women, excluding a portion of the population that could experience infertility.

Our main analysis showed an association between infertility and exposure to cadmium and our sensitivity analyses revealed associations between infertility and arsenic. Both metals are naturally occurring substances that can be found in air, water, and soil [83,84]. Research on the link between cadmium and arsenic and infertility remains limited [85,86,87]. A study of women in China found higher urinary cadmium levels in women who lived closer to a zinc mine, which was associated with difficulties in becoming pregnant and other pregnancy-related issues [85]. Another prospective study of 501 US couples reported a significant connection between high blood cadmium levels in the female partner and reduced fecundity [86]. Lastly, a study in Denmark examined occupational exposure to lead, mercury, and cadmium and found that exposed females to all of these metals had a higher likelihood of experiencing pregnancy-related issues, including conception delay and idiopathic infertility [87]. Studies in Taiwan and India have linked arsenic exposure to adverse reproductive outcomes [88,89]. In Taiwan, infertile women were found to have significantly higher blood arsenic levels compared to pregnant women, while in India, areas with arsenic exposure reported higher rates of stillbirth, recurrent miscarriage, and infertility compared to non-exposed areas [88,89]. A separate study observed no significant difference in blood arsenic concentration between women who became pregnant and those who did not [90]. The emerging evidence suggests that exposure to cadmium and arsenic may negatively impact female fertility. These findings highlight the potential reproductive risks of cadmium and arsenic, emphasizing the importance of reducing exposure to these metals to improve fertility outcomes.

Although the association between hexachlorobenzene (HCB) and self-reported infertility was not statistically significant after adjusting for confounders (likely due to the small sample size), its widespread use for pest and fungus control warrants attention, as HCB has been linked to reproductive health issues in other studies [91,92,93,94,95]. Although banned in the US since 1966, HCB is still produced abroad and generated as a byproduct of organic chemical production [96]. In the US, people are exposed to HCB primarily by consuming contaminated foods, such as fatty fish [96]. HCB is a persistent chemical that accumulates in adipose tissue, persisting for years [96]. A birth cohort study conducted in France showed that elevated cord blood levels of HCB, collected at birth as a proxy for in utero exposure, were associated with prolonged time to pregnancy [21]. Meanwhile, other studies suggest similar findings pointing to the chemical’s adverse reproductive effects [97,98].

Despite chlordane’s 1988 ban, our study shows continued exposure to oxychlordane, its metabolite and elevated odds of self-reported infertility in highly exposed individuals. Exposure in the US may result from inhalation, dermal contact with soil from termite-treated old homes, or consumption of chlordane-contaminated food or drinking water that was potentially tainted before the ban [99]. In the US, an estimated 52 million people live in chlordane-treated homes [99]. Additionally, HCB is a byproduct of manufacturing processes and the combustion of municipal waste [100]. Like HCB, chlordane residues accumulate in body fat, where it can be stored for a long time [99]. The limited research suggests reduced fertility in rats exposed to chlordane in their diet [101]. Although human studies on exposure to HCB and infertility are limited, some have observed decreased fertility [21,102]. Therefore, epidemiological studies investigating the reproductive effects of HCB and other pesticide compounds are warranted.

Oxidative stress, endocrine disruption, and systemic inflammation are likely mechanisms through which environmental chemicals can interfere with infertility. Environmental chemicals can trigger oxidative stress which can lead to ovarian aging, apoptosis in follicles, reduced follicle reserve, impaired follicle formation, and growth, ultimately affecting infertility [103,104,105]. Regulation of oxidative stress is an important factor in efforts to conceive, as evident through the use of antioxidant supplements [106]. Endocrine disruption plays a critical role in linking exposure to environmental chemicals and toxins to infertility by interfering with hormone systems, leading to hormonal imbalances; these disruptions can impair ovulation, menstrual cycles, and overall reproductive function, thereby increasing the risk of infertility [107,108]. Systemic inflammation, induced by exposure to environmental chemicals, can affect the reproductive tract leading to various impairments in reproductive function and thereby contributing to infertility [109,110].

The inverse associations between exposure to some PFAs are unexpected given the numerous studies suggesting positive associations [34,111]. One possible explanation is through a non-linear relationship, but we tested this and did not detect non-linearity in our analyses. Another possibility is that a sociodemographic variable or other third variables might confound this relationship, as individuals with different characteristics may have varying exposure levels and fertility outcomes. Additionally, potential exposure misclassification and/or the small sample size that contributed to environmental samples. Such findings should be further explored in future studies.

In general, the exposure disparities and associations with infertility for some chemicals are important public health concerns. Despite reductions in the environmental concentration of many hazardous chemicals due to various environmental health policies and changes in industrial practices, human exposure persists due to the persistent nature of these chemicals. This underscores the need for continuous monitoring and assessment of health risks with special attention to the sociodemographic disparities of exposures. The sociodemographic disparities in exposure to harmful chemicals also highlight the need for more advocacy in environmental justice principles [112]. Additionally, infertility due to environmental chemical exposure, which is often overlooked, can hinder the fundamental human right to have children if and when desired. Since 1948, international human rights efforts have affirmed an individual’s right to build families and make personal, informed choices about when and how many children to have [113]. Addressing chemicals’ impact on fertility is vital for ensuring family-building opportunities. Given population diversity, rising infertility rates, and complex environmental threats, equitable and sustainable exposure reduction efforts are necessary. While awaiting more studies to further understand the full extent of the health impacts of environmental chemicals, their negative health consequences are undeniable [114,115,116]. Yet, many reproductive-aged women are unaware of exposures and their impact on fertility [117]. Thus, it may be prudent that healthcare providers—through trusted partnerships—inform and advocate for safer chemical policies, as well as discuss potential health impacts with patients. Currently, healthcare providers seldom warn expectant or aspiring mothers about environmental hazards due to limited training, concerns about causing anxiety, and the perceived limited exposure-reduction options [118]. Studies have highlighted a significant gap in environmental health education within the US medical training curriculum and this deficiency raises concerns about the preparedness of future physicians to effectively manage growing environmental concerns and related illnesses [119,120,121].

At the personal level, approaches to reduce exposure include selecting fresh foods, minimizing processed and canned foods with plastic liners, adopting home habits like removing shoes indoors, and checking local air quality [122]. On the public health front, multidisciplinary collaboration among policymakers, clinicians, industry partners, researchers, governmental agencies, and community groups is crucial to developing effective mitigation and prevention strategies against widespread environmental chemicals. Meanwhile, consistent education and communication efforts regarding the role of environmental chemicals on health is needed [123]. People are inclined to act upon knowledge of risks but may hesitate when faced with conflicting information [123,124,125].

This study has several limitations. The cross-sectional nature and the lack of information on the timing of exposures and lack of infertility diagnosis limit our ability to infer the temporality of such a relationship. Second, infertility was based on a single question that was self-reported, leading to potential outcome misclassification. Although we defined people as infertile if they had actively been trying for 12 months AND were not able to conceive, this binary approach may unintentionally misclassify individuals. However, there is no reason to expect differential misclassification by exposure status, as people are typically unaware of their exposure level. Studies showed that self-reported infertility is an appropriate and valid measure with high specificity at 95% and sensitivity at 70% when compared against medical records [126]. Third, the way infertility is defined in this study may underestimate infertility for women ≥35 as the definition is different for this group. Fourth, we did not have data on male factors, although they contribute to about one-third of infertility cases [19]. Fifth, excluding participants without a fertility response reduced our sample by approximately 15%, which could impact generalizability. Although this exclusion was necessary, as fertility response defined eligibility, it may have excluded individuals potentially experiencing lower levels of fertility. In analyses comparing characteristics between those in the sample and those excluded, we found that those with a fertility response included a lower proportion of NH White individuals and a higher proportion of NH Asian individuals compared to the analytical sample. However, both groups were similar in terms of age, income and education. Sixth, since we classified exposures by the median, individuals with levels below fertility-impacting thresholds may have been included in the exposed group (and vice versa depending on the threshold). This potential misclassification may have distorted our findings. However, we also conducted sensitivity analyses using the 75th percentile as a threshold. This higher cut-off allows for a more stringent classification of ‘high exposure’ and provides additional confidence that our findings capture associations at levels more likely to impact fertility. Nevertheless, we note that because there are no established health thresholds for these chemicals, our cut-offs are arbitrary and may not accurately capture fertility effects. Lastly, the limited sample size hindered our ability to identify susceptible subgroups.

Despite the limitations, our study has notable strengths. First, the study is nationally representative and provides an analysis of the relationship between environmental chemicals and infertility in the US. Second, the use of biomarker data can help minimize exposure misclassification. Lastly, the multiple sensitivity analyses with different exposure metrics showed consistent findings, which was reassuring.

## 5. Conclusions

In a US nationally representative sample of reproductive-aged women, we found that high levels of exposure to pesticides, metals, and potentially some BFRs were associated with significantly higher odds of self-reported infertility. Furthermore, exposures to these chemicals varied across sociodemographic characteristics where marginalized groups are more burdened with exposure to environmental chemicals. Despite reductions in the environmental concentration of many hazardous chemicals including BFRs, certain metals, and pesticides, due to various environmental health policies and changes in industrial practices, human exposure to these substances continues. This ongoing exposure is also largely due to the persistent nature of these chemicals in the environment. This concern underscores the need for continuous monitoring and assessment of health risks with special attention to the sociodemographic disparities in exposures. The sociodemographic disparities in exposure to harmful chemicals also highlight the need for more advocacy in environmental justice principles, defined as equal protection in environmental and health laws [112]. Continued precautionary measures are crucial to protect equal rights to good health regardless of sociodemographic status. While awaiting larger studies to deepen our understanding of the profound effects of environmental chemicals on infertility, it is critical to continue and strengthen initiatives aimed at reducing exposure.

## Figures and Tables

**Table 1:** Individual characteristics of reproductive-aged women from the 2013–2016 National Health and Nutrition Examination Survey (NHANES) by infertility status (n = 2579).

Characteristics	All ^a^		Infertility ^a^		No Infertility ^a^		*p*-Value ^b^
	n = 2579	% and CI ^c^	n = 293	% and CI ^d^	n = 2286	% and CI ^d^	
Age (years)							<0.0001
18–29	1049	39.1 (36.3–42.0)	64	5.6 (4.0–7.2)	985	94.3 (92.7–95.9)	
30–39	751	30.1 (27.6–32.6)	98	14.5 (11.6–17.5)	653	85.4 (82.4–88.3)	
40–49	779	30.6 (28.3–33.0)	131	19.7 (16.0–23.4)	648	80.2 (76.5–83.9)	
Race/Ethnicity							0.22
NH White	842	57.3 (51.0–63.6)	111	13.8 (11.0–16.7)	731	86.1 (83.2–88.9)	
NH Black	551	13.0 (9.5–16.5)	64	12.2 (9.3–15.0)	487	87.7 (84.9–90.6)	
Hispanic	767	19.6 (15.0–24.3)	74	10.4 (8.2–12.5)	693	89.56 (87.4–91.7)	
NH Asian	310	6.1 (4.5–7.5)	30	9.6 (6.7–12.6)	280	90.3 (87.3–93.2)	
Other/Multi	109	3.7 (2.8–4.5)	14	11.8 (5.0–18.5)	95	88.1 (81.4–94.9)	
Education Level							0.25
Less than High School	467	13.3 (10.9–15.6)	40	9.2 (5.6–12.7)	427	90.7 (87.2–94.3)	
High School graduate/GED	557	18.9 (16.2–21.6)	56	11.5 (8.2–14.7)	501	88.4 (85.2–91.7)	
Some College/AA Degree	875	34.7 (32.0–37.5)	112	12.9 (10.5–15.4)	763	87.0 (84.5–89.4)	
College Graduate or more	676	32.8 (28.6–37.0)	84	14.3 (10.9–17.6)	592	85.6 (82.3–89.0)	
Missing	4	0.0 (0.0–0.1)	1	21.8 (0.0–63.3)	3	78.1 (36.6–100.0)	
Marital Status							<0.0001
Married/Cohabiting	1343	57.6 (54.1–61.1)	211	16.9 (14.2–19.7)	1132	83.0 (80.2–85.7)	
Single/Divorced/ Widow	956	36.5 (33.0–39.9)	78	7.6 (5.9–9.4)	878	92.3 (90.5–94.0)	
Missing	280	5.8 (4.7–6.9)	4	1.1 (0.0–2.4)	276	98.8 (97.5–100.0)	
Annual Family Income							0.0003
<$45k	1242	40.9 (37.5–44.3)	127	9.9 (7.6–12.2)	1115	90.0 (87.7–92.3)	
$45k–$100k	713	29.3 (26.8–31.9)	86	14.5 (11.1–17.8)	627	85.4 (82.1–88.8)	
≥$100k	434	23.6 (19.7–27.6)	67	16.6 (11.8–21.4)	367	83.3 (78.5–88.1)	
Missing	190	5.9 (4.5–7.3)	13	6.1 (3.2–9.0)	177	93.8 (90.9–96.7)	
Alcohol Use							0.23
Yes	1595	70.5 (66.6–74.4)	204	13.2 (11.3–15.2)	1391	86.7 (84.7–88.6)	
No	89	29.4 (25.5–33.3)	89	11.17 (8.2–14.0)	895	88.8 (85.9–91.7)	
General Health Status							0.35
Excellent/Very Good	992	44.9 (41.5–48.3)	104	11.9 (9.1–14.8)	888	88.0 (85.1–90.8)	
Good	1059	39.1 (36.4–41.8)	117	12.3 (9.8–14.8)	942	87.6 (85.1–90.1)	
Fair/Poor	528	15.9 (13.6–18.2)	72	15.3 (11.2–19.3)	456	84.6 (80.6–88.7)	
Smoking Status							0.69
Current	425	18.2 (16.3–20.0)	56	12.5 (8.9–16.1)	369	87.4 (83.8–91.0)	
Former	261	13.0 (10.5–15.5)	41	14.5 (9.9–19.0)	220	85.4 (80.9–90.0)	
Never	1893	68.7 (65.5–71.9)	196	12.3 (10.3–14.3)	1697	87.6 (85.6–89.6)	
Body Mass Index (units)							0.0018
Underweight/Normal (<25	980	39.7 (36.4–42.9)	91	10.0 (7.8–12.2)	889	89.9 (87.7–92.1)	
Overweight (25.0–29.99)	611	23.6 (21.5–25.8)	50	8.9 (5.6–12.2)	561	91.0 (87.7–94.3)	
Obese (30.0+)	988	36.5 (34.1–39.0)	152	17.8 (14.0–21.7)	836	82.1 (78.2–85.9)	

^a^ The sample size (*n*) is unweighted but the percentage (%) accounted for the complex sampling design. ^b^ *p*-values were obtained using Kruskal–Wallis tests and accounted for the complex sampling design. ^c^ Column percent. ^d^ Row percent.

**Table 2:** Logistic regression analysis estimating the associations between environmental chemicals and self-reported infertility, NHANES 2013–2016.

Environmental Classes	Environmental Chemicals	N	Odds Ratio (95% CI) ^a^		
			Model 1 ^b^	Model 2 ^c^	Model 3 ^d^
Brominated Fire Retardants (BFRs)	2,2’,4,4’,5,5’-Hexabromobiphenyl (PBB-153) (pg/g)	798	**2.09 (1.24–3.53)**	1.12 (0.54–2.33)	1.44 (0.73–2.83)
	2,4’-Tribromodiphenyl ether (PBDE-28) (pg/g)	798	1.12 (0.71–1.77)	0.99 (0.61–1.61)	0.91 (0.58–1.43)
	2,2’,4,4’-Tetrabromodiphenyl ether (PBDE-47) (pg/g)	798	1.15 (0.64–2.05)	1.09 (0.57–2.05)	1.05 (0.56–1.94)
	2,2’,4,4’,5-Pentabromodiphnyl ether (PBDE-99) (pg/g)	798	1.02 (0.63–1.64)	0.90 (0.50–1.60)	0.92 (0.54–1.58)
	2,2’,4,4’,6-Pentabromodiphyl ether (PBDE-100) (pg/g)	798	0.94 (0.51–1.77)	1.02 (0.55–1.90)	0.94 (0.50–1.74)
	2,2’,4,4’,5,5’-Hxbromodiphnyl ether (PBDE-153) (pg/g)	798	1.16 (0.60–2.24)	0.74 (0.32–1.71)	1.03 (0.51–2.05)
Volatile Organic Compounds (VOCs)	1,4-Dichlorobenzene (ng/mL)	1214	1.03 (0.68–1.54)	1.19 (0.85–1.67)	1.28 (0.86–1.90)
	Benzene (ng/mL)	1192	0.92 (0.58–1.45)	1.10 (0.61–1.97)	1.00 (0.61–1.62)
	Toluene (ng/mL)	1199	0.79 (0.51–1.23)	0.87 (0.50–1.51)	0.86 (0.53–1.38)
	Methyl-tert-butyl ether (MTBE) (ng/mL)	1153	0.73 (0.13–3.95)	0.71 (0.14–3.43)	0.73 (0.15–3.48)
Cotinine	Cotinine (ng/mL)	2476	0.83 (0.60–1.13)	1.02 (0.71–1.47)	1.02 (0.77–1.34)
Metals	Arsenic, total (ug/L)	880	1.28 (0.73–2.23)	1.29 (0.71–2.33)	1.29 (0.73–2.29)
	Cadmium (ug/L)	880	**2.09 (1.20–3.61)**	**1.88 (1.02–3.47)**	**1.84 (1.06–3.20)**
	Lead (ug/dL)	1263	1.02 (0.68–1.54)	0.83 (0.50–1.36)	0.78 (0.49–1.24)
	Mercury, total (ug/L)	1263	1.05 (0.67–1.65)	0.96 (0.56–1.65)	0.94 (0.59–1.49)
Pesticides	3-(Ethlycarbamoyl) benzoic acid (DEET acid) (ng/mL)	806	1.15 (0.69–1.91)	1.38 (0.79–2.38)	1.31 (0.76–2.27)
	Hexachlorobenzene (HCB) (pg/g)	798	**2.04 (1.05–3.98)**	1.58 (0.55–4.50)	1.48 (0.52–4.19)
	Oxychlordane (OXYCHLOR) (pg/g)	798	**2.04 (1.12–3.69)**	1.09 (0.49–2.41)	1.29 (0.63–3.07)
Environmental Phenols	Bisphenol A (ng/mL)	789	0.82 (0.45–1.50)	1.10 (0.54–2.25)	1.06 (0.57–1.96)
Per- and polyfluoroalkyl substances (PFAs)	n-perfluorooctanoic acid (n-PFOA) (ng/mL)	752	**0.52 (0.31–0.86)**	**0.46 (0.26–0.81)**	**0.51 (0.30–0.86)**
	n-perfluorooctane sulfonic acid (n-PFOS) (ng/mL)	752	**0.51 (0.28–0.95)**	**0.46 (0.24–0.88)**	**0.51 (0.26–0.97)**
	Perfluorohexane sulfonic acid (PFHxS) (ng/mL)	795	0.93 (0.54–1.63)	1.01 (0.55–1.84)	1.10 (0.60–1.99)
	Perfluorononanoic acid (PFNA) (ng/mL)	795	0.63 (0.31–1.29)	0.51 (0.24–1.09)	0.53 (0.25–1.14)

^a^ Cut-off at the 50th percentile. ^b^ Model 1 is an unadjusted model that accounts for complex probability weighting. ^c^ Model 2 adjusted for confounders identified by the directed acyclic graph including age, race, education level, annual family income, and smoking status. ^d^ Model 3 was fully adjusted for covariates that were associated with both exposure and outcome in exploratory analyses, including age and annual family income. Boldface indicates *p*-value < 0.05. Abbreviations: CI, confidence intervals.

**Table 3:** Sensitivity analysis: logistic regression analysis estimating the associations between environmental chemicals and infertility, NHANES 2013–2016.

Environmental Classes	Environmental Chemicals ^a^	N	Odds Ratio (95% CI) ^a^		
			Model 1 ^b^	Model 2 ^c^	Model 3 ^d^
Brominated Fire Retardants (BFRs)	2,2’,4,4’,5,5’-Hexabromobiphenyl (PBB-153) (pg/g)	798	1.82 (1.00–3.34)	1.08 (0.47–2.48)	1.25 (0.59–2.62)
	2,4,4’-Tribromodiphenyl ether (PBDE-28) (pg/g)	798	1.13 (0.64–2.01)	1.00 (0.54–1.87)	0.88 (0.48–1.59)
	2,2’,4,4’-Tetrabromodiphenyl ether (PBDE-47) (pg/g)	798	0.86 (0.42–1.72)	0.89 (0.41–1.90)	0.81 (0.40–1.64)
	2,2’,4,4’,5-Pentabromodiphnyl ether (PBDE-99) (pg/g)	798	0.90 (0.49–1.64)	0.79 (0.42–1.47)	0.79 (0.43–1.43)
	2,2’,4,4’,6-Pentabromodiphyl ether (PBDE-100) (pg/g)	798	0.99 (0.57–1.73)	0.95 (0.53–1.69)	0.90 (0.51–1.60)
	2,2’,4,4’,5,5’-Hxbromodiphnyl ether (PBDE-153) (pg/g)	798	1.36 (0.76–2.46)	0.86 (0.43–1.73)	1.20 (0.64–2.28)
Volatile Organic Compounds (VOCs)	1,4-Dichlorobenzene (ng/mL)	1214	1.20 (0.76–1.89)	1.44 (0.85–2.46)	1.33 (0.84–2.10)
	Benzene (ng/mL)	1192	0.76 (0.48–1.20)	0.78 (0.43–1.41)	0.76 (0.47–1.23)
	Toluene (ng/mL)	1199	0.68 (0.42–1.08)	0.69 (0.36–1.33)	0.68 (0.40–1.14)
	Methyl-tert-butyl ether (MTBE) (ng/mL)	1153	0.73 (0.13–3.95)	0.71 (0.14–3.43)	0.70 (0.13–3.72)
Cotinine	Cotinine (ng/mL)	2476	1.02 (0.74–1.40)	1.72 (0.99–2.99)	1.12 (0.82–1.54)
Metals	Arsenic, total (ug/L)	880	**1.83 (1.04–3.22)**	**1.88 (1.05–3.36)**	1.67 (0.94–2.96)
	Cadmium (ug/L)	880	1.43 (0.79–2.59)	1.03 (0.53–2.01)	1.02 (0.57–1.81)
	Lead (ug/dL)	1263	0.83 (0.60–1.51)	0.79 (0.46–1.35)	0.72 (0.46–1.12)
	Mercury, total (ug/L)	1263	1.05 (0.63–1.75)	0.93 (0.55–1.59)	0.91 (0.55–1.51)
Pesticides	3-(Ethlycarbamoyl) benzoic acid (DEET acid) (ng/mL)	806	0.82 (0.40–1.69)	0.87 (0.40–1.90)	0.86 (0.41–1.81)
	Hexachlorobenzene (HCB) (pg/g)	798	**1.70 (1.00–2.90)**	1.46 (0.57–3.72)	1.03 (0.50–2.09)
	Oxychlordane (OXYCHLOR) (pg/g)	798	**1.73 (1.05–2.85)**	0.62 (0.19–2.02)	0.80 (0.33–1.88)
Environmental Phenols	Bisphenol A (ng/mL)	789	1.08 (0.55–2.12)	1.52 (0.68–3.42)	1.15 (0.56–2.36)
Per- and Polyfluoroalkyl substances (PFAs)	n-perfluorooctanoic acid (n-PFOA) (ng/mL)	752	0.60 (0.31–1.17)	0.50 (0.22–1.10)	0.57 (0.29–1.14)
	n-perfluorooctane sulfonic acid (n-PFOS) (ng/mL)	752	0.57 (0.32–1.00)	**0.51 (0.28–0.92)**	**0.52 (0.30–0.89)**
	Perfluorohexane sulfonic acid (PFHxS) (ng/mL)	795	0.62 (0.32–1.22)	0.71 (0.34–1.48)	0.73 (0.36–1.46)
	Perfluorononanoic acid (PFNA) (ng/mL)	795	0.67 (0.29–1.53)	0.62 (0.25–1.54)	0.59 (0.25–1.40)

^a^ Cut-off at the 75th percentile. ^b^ Model 1 accounts for complex probability weighting. ^c^ Model 2 adjusted for confounder identified by the directed acyclic graph including age, race, education level, annual family income, and smoking status. ^d^ Model 3 was fully adjusted for covariates including age and alcohol use. Boldface indicates *p*-value < 0.05. Abbreviations: CI, confidence intervals.

## Data Availability

The NHANES data used in this study can be found at https://wwwn.cdc.gov/nchs/nhanes/Default.aspx (accessed on 20 June 2021).

## Supplementary Materials

The following supporting information can be downloaded at: https://www.mdpi.com/article/10.3390/ijerph21121541/s1, Figure S1: Directed acyclic graph to identify potential confounders; Table S1: Environmental chemicals measured in biological tissue of reproductive aged women, NHANES 2013-2015; Table S2: Environmental chemical distribution across age groups; Table S3: Environmental chemical distribution across race/ethnicity groups; Table S4: Environmental chemical distribution across education levels; Table S5: Environmental chemical distribution across annual family income levels; Table S6: Environmental chemical distribution across marital status; Table S7: Environmental chemical distribution across general health status; Table S8: Environmental chemical distribution across body mass index levels; Table S9: Environmental chemical distribution across smoking status; Table S10: Environmental chemical distribution across alcohol status; Table S11: Environmental chemical distribution across infertility status; Table S12: Logistic regression analysis estimating the associations between exposure score and infertility.
