# Supplementary material for: Exposure to Environmental Chemicals and Infertility Among US Reproductive-Aged Women
Source: Int J Environ Res Public Health. 2024 Nov 21;21(12):1541. doi: 10.3390/ijerph21121541 (PMC11675402; doi:10.3390/ijerph21121541)
Supplement: Supplementary file 1 [file ijerph-21-01541-s001.zip › ManuscriptSupplementalFigures_NoMarkUp_112024_VM.pdf]

International Journal of
Environmental Research and Public Health

MDPI

Manuscript Supplemental Figures

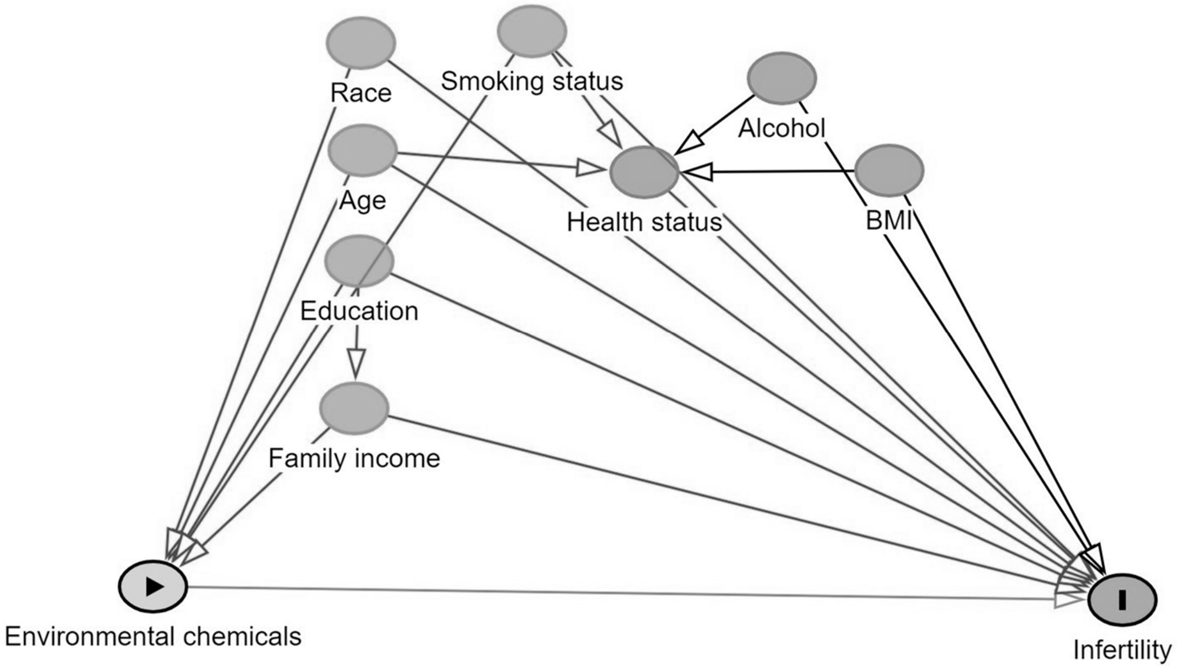

Figure S1. Directed acyclic graph to identify potential confounders.

Confounders = Race, Age, Education, Family Income, and Smoking Status

Ancestor of outcome (not confounder) = Health Status, Alcohol, BMI, and Infertility
