# Supplementary material for: Exposure to Environmental Chemicals and Infertility Among US Reproductive-Aged Women
Source: Int J Environ Res Public Health. 2024 Nov 21;21(12):1541. doi: 10.3390/ijerph21121541 (PMC11675402; doi:10.3390/ijerph21121541)
Supplement: Supplementary file 1 [file ijerph-21-01541-s001.zip › ManuscriptSupplementalTables_NoMarkUp_112024_VM.pdf]

International Journal of
Environmental Research and Public Health

MDPI

Supplemental Tables

Study title: Exposure to environmental chemicals and infertility among US reproductive-aged women

Authors: Valerie Martinez[1], Irene Yen[1], Camila Alvarez[2], Andrew Williams[3], Sandie Ha[1]

Affiliations:

1. Department of Public Health, School of Social Sciences, Humanities and Arts; Health Science Research Institute, University of California, Merced.
2. Department of Sociology; University of California, San Diego.
3. Public Health Program, School of Medicine and Health Sciences, University of North Dakota.

Table of Contents

Table S1. Environmental chemicals measured in biological tissue of reproductive aged women, NHANES 2013-2016. 2

Table S2. Environmental chemical distribution across age groups. 3

Table S3. Environmental chemical distribution across race/ethnicity groups. 5

Table S4. Environmental chemical distribution across education levels. 7

Table S5. Environmental chemical distribution across annual family income levels. 8

Table S6. Environmental chemical distribution across marital status. 10

Table S7. Environmental chemical distribution across general health status. 11

Table S8. Environmental chemical distribution across body mass index levels. 12

Table S9. Environmental chemical distribution across smoking status. 15

Table S10. Environmental chemical distribution across alcohol use. 16

Table S11. Environmental chemical distribution across infertility status. 18

Table S12. Logistic regression analysis estimating the associations between exposure score and infertility.19

Table S1. Environmental chemicals measured in biological tissue of reproductive aged women, NHANES 2013-2016.

Chemical Classes	Environmental Chemicals	Biospecimen	2013-2014 Maximum Limit of Detection (MLOD)/ Lower Limit of Detection (LLOD)	2015-2016 Maximum Limit of Detection (MLOD)/ Lower Limit of Detection (LLOD)	50th Percentile	75th Percentile
Brominated Flame Retardants (BFRs)	2,2',4,4',5,5'-Hexabromobiphenyl (PBB-153) (pg/g)	Serum	MLOD = 0.71 pg/g	MLOD = 1.1 pg/g	4.129	8.966
	2,4,4'-Tribromodiphenyl ether (PBDE-28) (pg/g)	Serum	MLOD = 0.71 pg/g	MLOD = 1.5 pg/g	4.164	6.083
	2,2',4,4'-Tetrabromodiphenyl ether (PBDE-47) (pg/g)	Serum	MLOD = 2.8 pg/g	MLOD = 1.3 pg/g	79.315	113.8
	2,2',4,4',5-Pentabromodiphnyl ether (PBDE-99) (pg/g)	Serum	MLOD = 1.2 pg/g	MLOD = 1.1 pg/g	14.39	21.97
	2,2',4,4',6-Pentabromodiphyl ether (PBDE-100) (pg/g)	Serum	MLOD = 0.71 pg/g	MLOD = 1.0 pg/g	15.71	22.75
	2,2',4,4',5,5'-Hxbromodiphnyl ether (PBDE-153) (pg/g)	Serum	MLOD = 0.71 pg/g	MLOD = 1.0 pg/g	37.8	58.04
Volatile Organic Compounds (VOCs)	1,4-Dichlorobenzene (ng/mL)	Blood	LLOD = 0.0400 ng/mL	LLOD = 0.040 ng/mL	0.0283	0.112
	Benzene (ng/mL)	Blood	LLOD = 0.0240 ng/mL	LLOD = 0.024 ng/mL	0.017	0.37
	Toluene (ng/mL)	Blood	LLOD = 0.0250 ng/mL	LLOD = 0.025 ng/mL	0.066	0.122
	Methyl-tert-butyl ether (MTBE) (ng/mL)	Blood	LLOD = 0.0100 ng/mL	LLOD = 0.010 ng/mL	0.0071	0.0071
Cotinine	Cotinine (ng/mL)	Serum	LLOD = 0.015 ng/mL	LLOD = 0.015 ng/mL	0.032	0.8975
Metals	Arsenic, total (ug/L)	Urine	LLOD = 0.26 ug/L	LLOD = 0.26 ug/L	5.7	11.1
	Cadmium (ug/L)	Urine	LLOD = 0.036 ug/L	LLOD = 0.036 ug/L	0.132	0.275

	Lead (ug/dL)	Blood	LLOD = 0.07 ug/dL	LLOD = 0.07 ug/dL	0.58	0.87
	Mercury, total (ug/L)	Blood	LLOD = 0.28 ug/L	LLOD = 0.28 ug/L	0.65	1.36
Pesticides	3-(Ethlycarbamoyl) benzoic acid (DEET acid) (ng/mL)	Urine	LLOD = 0.4750 ug/L	LLOD = 0.20 ug/L	1.94	7.37
	Hexachlorobenzene (HCB) (pg/g)	Serum	MLOD = 3.5 pg/g	MLOD = 3.5 pg/g	40.185	50.81
	Oxychlordane (OXYCHLOR) (pg/g)	Serum	MLOD = 3.5 pg/g	MLOD = 3.5 pg/g	17.98	37.06
Environmental Phenols	Bisphenol A (ng/mL)	Urine	LLOD = 0.2 ng/mL	LLOD = 0.2 ng/mL	1.3	2.6
Per- and polyfluoroalkyl substances (PFAs)	n-perfluorooctanoic acid (n-PFOA) (ng/mL)	Serum	LLOD = 0.1 ng/mL	LLOD = 0.10 ng/mL	1.1	1.6
	n-perfluorooctane sulfonic acid (n-PFOS) (ng/mL)	Serum	LLOD = 0.1 ng/mL	LLOD = 0.10 ng/mL	2.2	3.4
	Perfluorohexane sulfonic acid (PFHxS) (ng/mL)	Serum	LLOD = 0.1 ng/mL	LLOD = 0.1 ng/mL	0.6	1
	Perfluorononanoic acid (PFNA) (ng/mL)	Serum	LLOD = 0.1 ng/mL	LLOD = 0.1 ng/mL	0.5	0.7

Table S2. Environmental chemical distribution across age groups.

**Environmental Classes**	**Environmental Chemicals**	**Ages 18-29**	**Ages 30-39**	**Ages 40-49**	***p*-value**
		**Median (Range)**	**Median (Range)**	**Median (Range)**	
Brominated Flame Retardants (BFRs)		N=320	N=231	N=247	
	2,2',4,4',5,5'-Hexabromobiphenyl (PBB-153) (pg/g)	3.04(2.79-3.32)	3.91(3.46-4.42)	15.28(12.38-18.85)	**<.0001**
	2,4,4'-Tribromodiphenyl ether (PBDE-28) (pg/g)*	3.58(3.40-3.77)	3.56(3.33-3.81)	5.18(4.74-5.66)	**<.0001**
	2,2',4,4'-Tetrabromodiphenyl ether (PBDE-47) (pg/g)*	73.54(68.77-78.65)	67.59(63.02-72.50)	88.67(81.51-96.46)	**<.0001**
	2,2',4,4',5-Pentabromodiphnyl ether (PBDE-99) (pg/g)*	14.46(13.23-15.81)	12.41(11.36-13.55)	16.89(15.43-18.49)	**<.0001**
	2,2',4,4',6-Pentabromodiphyl ether (PBDE-100) (pg/g)*	16.14(14.90-17.47)	14.86(13.74-16.06)	18.62(16.86-20.58)	**<.0001**
	2,2',4,4',5,5'-Hxbromodiphnyl ether (PBDE-153) (pg/g)*	42.85(39.76-46.19)	41.43(37.74-45.49)	58.25(52.14-65.08)	**<.0001**
		N=485	N=368	N=361	

**Environmental Classes**	**Environmental Chemicals**	**Ages 18-29**	**Ages 30-39**	**Ages 40-49**	***p*-value**
		**Median (Range)**	**Median (Range)**	**Median (Range)**	
Volatile Organic Compounds (VOCs)	1,4-Dichlorobenzene (ng/mL)	0.06(0.05-0.07)	0.05(0.04-0.06)	0.05(0.04-0.07)	0.0917
		N=475	N=356	N=361	
	Benzene (ng/mL)	0.02(0.02-0.03)	0.03(0.02-0.03)	0.02(0.02-0.03)	0.1813
		N=476	N=361	N=362	
	Toluene (ng/mL)	0.07(0.06-0.08)	0.09(0.07-0.10)	0.08(0.07-0.09)	0.6090
		N=464	N=347	N=342	
	Methyl-tert-butyl ether (MTBE) (ng/mL)	0.007(0.007-0.007)	0.007(0.007-0.007)	0.007(0.007-0.007)	0.6255
Cotinine		N=992	N=728	N=756	
	Cotinine (ng/mL)	0.26(0.18-0.37)	0.17(0.12-0.24)	0.12(0.08-0.17)	**<.0001**
Metals		N=358	N=260	N=262	
	Arsenic, total (ug/L)	5.58(4.82-6.44)	5.41(4.60-6.36)	6.32(5.12-7.80)	0.3152
		N=358	N=260	N=262	
	Cadmium (ug/L)	0.08(0.07-0.09)	0.11(0.09-0.14)	0.18(0.15-0.22)	**<.0001**
		N=502	N=378	N=383	
	Lead (ug/dL)	0.48(0.44-0.53)	0.60(0.55-0.64)	0.73(0.67-0.79)	**<.0001**
	Mercury, total (ug/L)*	0.58(0.52-0.65)	0.77(0.69-0.86)	0.82(0.72-0.94)	**<.0001**
Pesticides		N=307	N=230	N=269	
	3-(Ethlycarbamoyl) benzoic acid (DEET acid) (ng/mL)	2.84(2.12-3.79)	2.61(1.72-3.97)	2.06(1.32-3.23)	**0.0420**
		N=320	N=231	N=247	
	Hexachlorobenzene (HCB) (pg/g)	35.43(34.30-36.59)	37.73(36.61-38.89)	56.16(54.31-58.08)	**<.0001**
	Oxychlordane (OXYCHLOR) (pg/g)*	14.29(13.57-15.05)	17.01(16.11-17.95)	48.88(46.19-51.73)	**<.0001**
Environmental Phenols		N=316	N=229	N=244	
	Bisphenol A (ng/mL)	1.19(0.95-1.49)	1.04(0.73-1.49)	0.97(0.56-1.67)	**0.0037**
		N=300	N=217	N=235	

Environmental Classes	Environmental Chemicals	Ages 18-29	Ages 30-39	Ages 40-49	*p*-value
		**Median (Range)**	**Median (Range)**	**Median (Range)**	
Per- and polyfluoroalkyl substances (PFAs)	n-perfluorooctanoic acid (n-PFOA) (ng/mL)	1.35(1.12-1.62)	1.00(0.82-1.23)	1.50(1.12-2.01)	**<.0001**
	n-perfluorooctane sulfonic acid (n-PFOS) (ng/mL)*	2.73(2.31-3.22)	1.99(1.57-2.52)	2.70(2.21-3.30)	**0.0004**
		N=318	N=230	N=247	
	Perfluorohexane sulfonic acid (PFHxS) (ng/mL)	0.71(0.57-0.89)	0.60(0.48-0.74)	0.60(0.46-0.78)	**<.0001**
	Perfluorononanoic acid (PFNA) (ng/mL)*	0.37(0.33-0.42)	0.36(0.28-0.46)	0.50(0.41-0.60)	**<.0001**

[1]*p*-values were obtained using Kruskal-Wallis Test.

[2]Boldface indicates p-value <0.05.

[*]Chemicals without n's have the same n's as the chemical listed directly above.

Table S3. Environmental chemical distribution across race/ethnicity groups.

Environmental Classes	Environmental Chemicals	NH Whites	NH Black	Hispanic	NH Asians	Other/ Multi	*p*-value
		**Median (Range)**	**Median (Range)**	**Median (Range)**	**Median (Range)**	**Median (Range)**	
Brominated Flame Retardants (BFRs)		N=261	N=185	N=216	N=101	N=35	
	2,2',4,4',5,5'-Hexabromobiphenyl (PBB-153) (pg/g)	6.71(5.62-8.02)	4.55(3.57-5.79)	4.13(3.78-4.50)	3.16(2.74-3.64)	2.49(2.06-2.99)	**<.0001**
	2,4,4'-Tribromodiphenyl ether (PBDE-28) (pg/g)*	3.94(3.70-4.20)	3.83(3.46-4.24)	4.20(3.79-4.66)	3.87(3.44-4.36)	5.19(4.59-5.87)	**0.0401**
	2,2',4,4'-Tetrabromodiphenyl ether (PBDE-47) (pg/g)*	75.66(70.83-80.83)	84.98(77.39-93.31)	75.16(67.67-83.47)	55.77(49.88-62.36)	101.55(83.04-124.18)	**<.0001**
	2,2',4,4',5-Pentabromodiphnyl ether (PBDE-99) (pg/g)*	14.53(13.36-15.81)	16.74(15.09-18.57)	13.89(12.30-15.69)	9.95(8.97-11.03)	20.68(14.40-29.69)	**<.0001**
	2,2',4,4',6-Pentabromodiphyl ether (PBDE-100) (pg/g)*	16.36(15.09-17.74)	20.27(18.69-21.99)	15.88(14.66-17.21)	11.62(10.31-13.10)	18.74(16.57-21.20)	**<.0001**
	2,2',4,4',5,5'-Hxbromodiphnyl ether (PBDE-153) (pg/g) *	58.35(54.57-62.39)	43.78(40.72-47.06)	30.95(28.99-33.05)	24.30(22.06-26.76)	41.45(36.28-47.35)	**<.0001**
Volatile Organic Compounds (VOCs)		N=407	N=236	N=386	N=136	N=49	
	1,4-Dichlorobenzene (ng/mL)	0.04(0.03-0.04)	0.12(0.08-0.17)	0.09(0.07-0.13)	0.05(0.04-0.07)	0.07(0.05-0.09)	**<.0001**
		N=398	N=233	N=383	N=130	N=48	
	Benzene (ng/mL)	0.03(0.02-0.03)	0.03(0.02-0.03)	0.02(0.02-0.02)	0.02(0.01-0.02)	0.03(0.02-0.04)	**<.0001**
		N=400	N=233	N=388	N=133	N=45	

	Toluene (ng/mL)	0.09(0.08-0.10)	0.08(0.07-0.10)	0.06(0.05-0.07)	0.06(0.05-0.06)	0.08(0.06-0.11)	**<.0001**
		N=379	N=219	N=374	N=134	N=47	
	Methyl-tert-butyl ether (MTBE) (ng/mL)	0.007(0.007-0.007)	0.007(0.007-0.007)	0.007(0.007-0.007)	0.007(0.007-0.007)	0.007(0.006-0.007)	**0.0290**
Cotinine		N=816	N=517	N=744	N=297	N=102	
	Cotinine (ng/mL)	0.23(0.17-0.31)	0.59(0.38-0.92)	0.04(0.03-0.06)	0.05(0.03-0.07)	0.55(0.21-1.40)	**<.0001**
Metals		N=293	N=167	N=282	N=104	N=34	
	Arsenic, total (ug/L)	4.98(4.35-5.70)	8.74(7.30-10.47)	5.86(4.99-6.87)	9.37(7.06-12.43)	4.92(3.67-6.60)	**<.0001**
		N=293	N=167	N=282	N=104	N=34	
	Cadmium (ug/L)	0.10(0.08-0.11)	0.20(0.16-0.25)	0.12(0.11-0.13)	0.15(0.13-0.18)	0.09(0.07-0.13)	**<.0001**
		N=427	N=250	N=396	N=139	N=51	
	Lead (ug/dL)	0.56(0.52-0.61)	0.58(0.53-0.63)	0.55(0.52-0.59)	0.89(0.79-0.99)	0.70(0.64-0.76)	**<.0001**
	Mercury, total (ug/L)*	0.65(0.58-0.72)	0.69(0.61-0.77)	0.68(0.61-0.76)	1.55(1.21-1.98)	0.90(0.76-1.07)	**<.0001**
Pesticides		N=268	N=184	N=229	N=96	N=29	
	3-(Ethlycarbamoyl) benzoic acid (DEET acid) (ng/mL)	2.66(1.75-4.03)	3.14(2.22-4.44)	2.36(1.59-3.50)	1.16(0.66-2.04)	2.19(1.21-3.95)	**<.0001**
		N=261	N=185	N=216	N=101	N=35	
	Hexachlorobenzene (HCB) (pg/g)	41.91(40.65-43.21)	33.69(31.86-35.62)	43.93(41.78-46.20)	54.64(49.57-60.22)	35.30(33.32-37.38)	**<.0001**
	Oxychlordane (OXYCHLOR) (pg/g)*	25.18(23.38-27.11)	19.42(17.04-22.13)	17.07(15.65-18.62)	19.16(16.70-21.98)	16.71(14.47-19.30)	**<.0001**
Environmental Phenols		N=257	N=182	N=215	N=101	N=34	
	Bisphenol A (ng/mL)	1.03(0.74-1.44)	1.62(1.35-1.94)	1.08(0.80-1.45)	0.70(0.52-0.94)	0.80(0.55-1.17)	**<.0001**
		N=247	N=174	N=203	N=94	N=34	
Per- and polyfluoroalkyl substances (PFAs)	n-perfluorooctanoic acid (n-PFOA) (ng/mL)	1.31(1.13-1.52)	0.78(0.70-0.88)	0.84(0.76-0.93)	1.41(1.29-1.54)	1.05(0.82-1.36)	**<.0001**
	n-perfluorooctane sulfonic acid (n-PFOS) (ng/mL)*	2.18(1.98-2.41)	2.23(1.81-2.74)	1.67(1.45-1.93)	3.82(3.09-4.72)	2.21(1.52-3.21)	**<.0001**
		N=260	N=185	N=215	N=100	N=35	
	Perfluorohexane sulfonic acid (PFHxS) (ng/mL)	0.77(0.63-0.94)	0.39(0.30-0.51)	0.52(0.43-0.63)	0.65(0.59-0.72)	0.55(0.33-0.89)	**<.0001**
	Perfluorononanoic acid (PFNA) (ng/mL)*	0.39(0.34-0.45)	0.38(0.32-0.47)	0.36(0.31-0.42)	0.76(0.63-0.92)	0.38(0.27-0.55)	**<.0001**

[1]*p*-values were obtained using Kruskal-Wallis Test.

[2]Boldface indicates p-value <0.05.

*Chemicals without n's have the same n's as the chemical listed directly above.

Table S4. Environmental chemical distribution across education levels.

**Environmental Classes**	**Environmental Chemicals**	**Less than High School**	**Highschool Graduate**	**Some college or AA degree**	**College graduate or more**	***p*-value**
		**Median (Range)**	**Median (Range)**	**Median (Range)**	**Median (Range)**	
Brominated Flame Retardants (BFRs)		N=149	N=160	N=278	N=211	
	2,2',4,4',5,5'-Hexabromobiphenyl (PBB-153) (pg/g)	4.14(3.29-5.20)	4.57(3.80-5.49)	5.28(4.43-6.29)	6.70(5.51-8.14)	**<.0001**
	2,4,4'-Tribromodiphenyl ether (PBDE-28) (pg/g)*	4.61(4.31-4.94)	3.87(3.54-4.24)	4.02(3.76-4.29)	3.82(3.55-4.10)	0.1188
	2,2',4,4'-Tetrabromodiphenyl ether (PBDE-47) (pg/g)*	86.68(81.03-92.72)	76.33(68.76-84.74)	77.33(71.28-83.89)	70.08(65.46-70.02)	**0.0023**
	2,2',4,4',5-Pentabromodiphnyl ether (PBDE-99) (pg/g)*	16.58(5.27-18.01)	14.76(12.86-16.90)	15.17(13.73-16.75)	12.88(11.82-14.03)	**<.0001**
	2,2',4,4',6-Pentabromodiphyl ether (PBDE-100) (pg/g)*	18.37(17.15-19.66)	16.78(14.89-18.91)	45.73(41.65-50.21)	15.50(14.36-16.73)	**0.0003**
	2,2',4,4',5,5'-Hxbromodiphnyl ether (PBDE-153) (pg/g)*	41.15(36.07-46.94)	43.79(38.54-49.77)	45.73(41.65-50.25)	51.83(46.62-57.61)	0.1247
Volatile Organic Compounds (VOCs)		N=230	N=264	N=401	N=317	
	1,4-Dichlorobenzene (ng/mL)	0.07(0.06-0.10)	0.06(0.05-0.09)	0.05(0.04-0.07)	0.04(0.03-0.05)	**<.0001**
		N=227	N=258	N=396	N=309	
	Benzene (ng/mL)	0.03(0.03-0.04)	0.03(0.02-0.03)	0.03(0.02-0.03)	0.02(0.02-0.02)	**0.0001**
		N=229	N=260	N=397	N=312	
	Toluene (ng/mL)	0.10(0.08-0.11)	0.08(0.07-0.10)	0.09(0.07-0.11)	0.07(0.06-0.08)	**0.0003**
		N=216	N=254	N=381	N=300	
	Methyl-tert-butyl ether (MTBE) (ng/mL)	0.007(0.007-0.007)	0.007(0.007-0.007)	0.007(0.007-0.007)	0.007(0.007-0.007)	0.5732
Cotinine		N=455	N=526	N=842	N=650	
	Cotinine (ng/mL)	0.51(0.26-0.99)	0.47(0.30-0.75)	0.29(0.22-0.39)	0.04(0.03-0.05)	**<.0001**
Metals		N=164	N=193	N=291	N=231	
	Arsenic, total (ug/L)	4.94(4.00-6.10)	6.14(5.26-7.17)	5.96(5.02-7.08)	5.66(4.67-6.87)	0.0780
	Cadmium (ug/L)*	0.14(0.11-0.16)	0.11(0.09-0.14)	0.12(0.11-0.14)	0.10(0.08-0.12)	0.4749
		N=236	N=276	N=415	N=334	
	Lead (ug/dL)	0.62(0.57-0.67)	0.49(0.44-0.55)	0.57(0.53-0.61)	0.64(0.58-0.72)	**<.0001**

	Mercury, total (ug/L)*	0.55(0.48-0.63)	0.56(0.48-0.65)	0.66(0.60-0.74)	0.94(0.82-1.08)	**<.0001**
Pesticides		N=134	N=165	N=289	N=217	
	3-(Ethlycarbamoyl) benzoic acid (DEET acid) (ng/mL)	3.13(2.12-4.62)	2.23(1.42-3.51)	3.23(2.39-4.38)	1.85(1.21-2.81)	**0.0399**
		N=149	N=160	N=278	N=211	
	Hexachlorobenzene (HCB) (pg/g)	42.49(40.71-44.36)	40.22(38.39-42.13)	39.52(38.09-40.99)	44.15(42.40-45.97)	**<.0001**
	Oxychlordane (OXYCHLOR) (pg/g)*	20.28(17.93-22.93)	19.74(17.62-22.12)	20.78(19.18-22.53)	25.38(23.28-27.67)	**<.0001**
Environmental Phenols		N=148	N=156	N=275	N=210	
	Bisphenol A (ng/mL)	1.10(0.80-1.51)	1.29(0.96-1.73)	1.44(1.05-1.99)	0.75(0.58-0.97)	**<.0001**
Per- and polyfluoroalkyl substances (PFAs)		N=135	N=153	N=266	N=198	
	n-perfluorooctanoic acid (n-PFOA) (ng/mL)	0.90(0.76-1.07)	1.06(0.79-1.43)	1.07(0.96-1.20)	1.32(1.14-1.53)	**<.0001**
	n-perfluorooctane sulfonic acid (n-PFOS) (ng/mL)*	2.02(1.78-2.30)	1.95(1.69-2.24)	2.30(2.06-2.56)	2.19(1.84-2.60)	0.0838
		N=149	N=159	N=278	N=209	
	Perfluorohexane sulfonic acid (PFHxS) (ng/mL)	0.53(0.38-0.73)	0.67(0.50-0.90)	0.60(0.47-0.77)	0.69(0.56-0.84)	0.2962
	Perfluorononanoic acid (PFNA) (ng/mL)*	0.34(0.26-0.43)	0.41(0.36-0.47)	0.41(0.36-0.46)	0.42(0.34-0.51)	**0.0022**

[1]*p*-values were obtained using Kruskal-Wallis Test.

[2]Boldface indicates p-value <0.05.

[*]Chemicals without n's have the same n's as the chemical listed directly above.

Table S5. Environmental chemical distribution across annual family income levels.

**Environmental Classes**	**Environmental Chemicals**	**<$45k**	**$45k-$99k**	**≥$100k**	***p*-value**
		**Median (Range)**	**Median (Range)**	**Median (Range)**	
Brominated Flame Retardants (BFRs)		N=384	N=227	N=130	
	2,2',4,4',5,5'-Hexabromobiphenyl (PBB-153) (pg/g)	4.49(3.93-5.12)	5.18(4.51-5.94)	7.39(5.74-9.52)	**0.0454**
	2,4,4'-Tribromodiphenyl ether (PBDE-28) (pg/g)*	4.06(3.82-4.30)	3.86(3.62-4.12)	4.17(3.72-4.66)	0.6113
	2,2',4,4'-Tetrabromodiphenyl ether (PBDE-47) (pg/g)*	78.26(73.60-83.22)	73.98(69.29-78.99)	76.29(67.72-85.96)	0.1796
	2,2',4,4',5-Pentabromodiphnyl ether (PBDE-99) (pg/g)*	15.30(14.10-16.60)	13.97(12.92-15.10)	14.23(12.36-16.38)	**0.0180**
	2,2',4,4',6-Pentabromodiphyl ether (PBDE-100) (pg/g)*	17.15(15.89-18.50)	15.77(14.81-16.78)	16.45(14.49-18.68)	0.0557

	2,2',4,4',5,5'-Hxbromodiphnyl ether (PBDE-153) (pg/g)*	44.52(41.59-47.65)	45.91(42.18-49.96)	53.31(47.64-59.65)	0.1384
Volatile Organic Compounds (VOCs)		N=568	N=351	N=205	
	1,4-Dichlorobenzene (ng/mL)	0.07(0.06-0.10)	0.07(0.06-0.10)	0.04(0.03-0.04)	**<.0001**
		N=559	N=343	N=200	
	Benzene (ng/mL)	0.03(0.02-0.04)	0.03(0.02-0.04)	0.02(0.02-0.02)	**<.0001**
		N=563	N=344	N=202	
	Toluene (ng/mL)	0.09(0.07-0.10)	0.09(0.07-0.10)	0.07(0.06-0.09)	**0.0327**
		N=541	N=337	N=193	
	Methyl-tert-butyl ether (MTBE) (ng/mL)	0.007(0.007-0.007)	0.007(0.007-0.007)	0.007(0.007-0.007)	0.3938
Cotinine		N=1182	N=700	N=414	
	Cotinine (ng/mL)	0.57(0.42-0.76)	0.11(0.08-0.17)	0.04(0.03-0.06)	**<.0001**
Metals		N=408	N=252	N=156	
	Arsenic, total (ug/L)	5.77(4.90-6.78)	6.29(5.57-7.12)	4.99(4.00-6.22)	0.1552
	Cadmium (ug/L)*	0.12(0.11-0.13)	0.12(0.10-0.15)	0.09(0.07-0.12)	0.7357
		N=600	N=358	N=214	
	Lead (ug/dL)	0.60(0.55-0.65)	0.53(0.48-0.58)	0.62(0.54-0.71)	**0.0042**
	Mercury, total (ug/L)*	0.63(0.57-0.60)	0.63(0.54-0.74)	0.95(0.80-1.11)	**<.0001**
Pesticides		N=391	N=228	N=125	
	3-(Ethlycarbamoyl) benzoic acid (DEET acid) (ng/mL)	3.20(2.49-4.12)	1.92(1.35-2.69)	2.24(1.31-3.83)	0.0897
		N=384	N=227	N=130	
	Hexachlorobenzene (HCB) (pg/g)	39.03(37.68-40.43)	42.22(40.22-44.33)	45.17(42.95-47.50)	**<.0001**
	Oxychlordane (OXYCHLOR) (pg/g)*	19.23(17.87-20.70)	21.78(19.92-23.82)	28.42(25.38-31.81)	**<.0001**
Environmental Phenols		N=378	N=225	N=130	
	Bisphenol A (ng/mL)	1.29(1.04-1.60)	0.84(0.59-1.18)	1.08(0.74-1.58)	**0.0010**
Per- and polyfluoroalkyl substances (PFAs)		N=357	N=220	N=121	
	n-perfluorooctanoic acid (n-PFOA) (ng/mL)	0.92(0.82-1.02)	1.16(0.96-1.42)	1.52(1.35-1.73)	**<.0001**
	n-perfluorooctane sulfonic acid (n-PFOS) (ng/mL)*	1.97(1.74-2.23)	2.15(1.87-2.48)	2.43(2.07-2.84)	**0.0027**
		N=383	N=227	N=129	
	Perfluorohexane sulfonic acid (PFHxS) (ng/mL)	0.61(0.48-0.77)	0.63(0.49-0.83)	0.66(0.54-0.79)	**0.0301**
	Perfluorononanoic acid (PFNA) (ng/mL)*	0.35(0.30-0.41)	0.44(0.35-0.54)	0.43(0.39-0.48)	**<.0001**

[1]*p*-values were obtained using Kruskal-Wallis Test.

[2]Boldface indicates p-value <0.05.

[*]Chemicals without n's have the same n's as the chemical listed directly above.

Table S6. Environmental chemical distribution across marital status.

Environmental Classes	Environmental Chemicals	Single/Divorced/ Widow	Married/Cohabiting	*p*-value
		**Median (Range)**	**Median (Range)**	
Brominated Flame Retardants (BFRs)		N=305	N=411	
	2,2',4,4',5,5'-Hexabromobiphenyl (PBB-153) (pg/g)	4.89(4.23-5.65)	6.42(5.38-7.64)	**0.0142**
	2,4,4'-Tribromodiphenyl ether (PBDE-28) (pg/g)*	3.85(3.59-4.12)	4.11(3.90-4.34)	0.1269
	2,2',4,4'-Tetrabromodiphenyl ether (PBDE-47) (pg/g)*	75.05(69.90-80.59)	75.35(71.37-79.56)	0.7735
	2,2',4,4',5-Pentabromodiphnyl ether (PBDE-99) (pg/g)*	14.51(13.28-15.85)	14.18(13.30-15.13)	0.9259
	2,2',4,4',6-Pentabromodiphyl ether (PBDE-100) (pg/g)*	16.31(14.90-17.85)	16.43(15.42-17.50)	0.9573
	2,2',4,4',5,5'-Hxbromodiphnyl ether (PBDE-153) (pg/g)*	45.40(41.06-50.20)	48.31(44.78-52.11)	0.4369
Volatile Organic Compounds (VOCs)		N=454	N=627	
	1,4-Dichlorobenzene (ng/mL)	0.06(0.05-0.08)	0.05(0.04-0.06)	**0.0007**
		N=444	N=616	
	Benzene (ng/mL)	0.03(0.02-0.03)	0.02(0.02-0.03)	**0.0252**
		N=445	N=624	
	Toluene (ng/mL)	0.09(0.08-0.11)	0.07(0.06-0.09)	**0.0076**
		N=429	N=594	
	Methyl-tert-butyl ether (MTBE) (ng/mL)	0.007(0.007-0.007)	0.007(0.007-0.007)	0.0636
Cotinine		N=919	N=1,302	
	Cotinine (ng/mL)	0.47(0.36-0.62)	0.10(0.07-0.13)	**<.0001**
Metals		N=321	N=458	
	Arsenic, total (ug/L)	6.48(5.67-7.42)	5.38(4.64-6.23)	**0.0340**
	Cadmium (ug/L)*	0.12(0.10-0.14)	0.12(0.10-0.13)	0.7150
		N=471	N=655	
	Lead (ug/dL)	0.60(0.56-0.64)	0.60(0.55-0.65)	0.5014
	Mercury, total (ug/L)*	0.72(0.65-0.81)	0.73(0.66-0.80)	0.8167
Pesticides		N=306	N=430	
	3-(Ethlycarbamoyl) benzoic acid (DEET acid) (ng/mL)	3.01(2.13-4.23)	2.18(1.50-3.17)	0.1091
		N=305	N=411	
	Hexachlorobenzene (HCB) (pg/g)	39.42(38.14-40.74)	44.25(42.69-45.86)	**<.0001**
	Oxychlordane (OXYCHLOR) (pg/g)*	20.96(19.46-22.58)	24.52(22.52-26.69)	**0.0439**

Environmental Phenols		N=298	N=409	
	Bisphenol A (ng/mL)	1.32(1.12-1.56)	0.93(0.68-1.27)	**0.0012**
Per- and polyfluoroalkyl substances (PFAs)		N=294	N=385	
	n-perfluorooctanoic acid (n-PFOA) (ng/mL)	1.21(1.09-1.35)	1.06(0.93-1.22)	**0.0378**
	n-perfluorooctane sulfonic acid (n-PFOS) (ng/mL)*	2.17(1.93-2.44)	2.11(1.88-2.36)	0.8966
		N=305	N=409	
	Perfluorohexane sulfonic acid (PFHxS) (ng/mL)	0.70(0.57-0.85)	0.59(0.49-0.71)	**0.0035**
	Perfluorononanoic acid (PFNA) (ng/mL)*	0.42(0.36-0.49)	0.40(0.35-0.45)	0.4198

[1]*p*-values were obtained using Kruskal-Wallis Test.

[2]Boldface indicates p-value <0.05.

[*]Chemicals without n's have the same n's as the chemical listed directly above.

Table S7. Environmental chemical distribution across general health status.

**Environmental Classes**	**Environmental Chemicals**	**Excellent/Very Good**	**Good**	**Fair/Poor**	***p*-value**
		**Median (Range)**	**Median (Range)**	**Median (Range)**	
Brominated Flame Retardants (BFRs)		N=290	N=349	N=159	
	2,2',4,4',5,5'-Hexabromobiphenyl (PBB-153) (pg/g)	5.90(4.77-7.29)	5.08(4.26-5.96)	4.87(4.20-5.64)	0.4835
	2,4,4'-Tribromodiphenyl ether (PBDE-28) (pg/g)*	4.04(3.79-4.30)	3.87(3.60-4.15)	4.28(3.93-4.65)	0.4580
	2,2',4,4'-Tetrabromodiphenyl ether (PBDE-47) (pg/g)*	76.36(71.37-81.69)	73.49(67.92-79.51)	81.36(74.51-88.83)	0.3945
	2,2',4,4',5-Pentabromodiphnyl ether (PBDE-99) (pg/g)*	14.27(13.02-15.64)	14.27(13.10-15.55)	15.70(14.41-17.09)	0.2113
	2,2',4,4',6-Pentabromodiphyl ether (PBDE-100) (pg/g)*	16.42(15.06-17.90)	16.04(14.71-17.49)	17.65(16.32-19.09)	0.0554
	2,2',4,4',5,5'-Hxbromodiphnyl ether (PBDE-153) (pg/g)*	50.62(46.44-55.16)	43.87(39.74-48.43)	43.12(39.22-47.41)	0.1089
Volatile Organic Compounds (VOCs)		N=473	N=483	N=258	
	1,4-Dichlorobenzene (ng/mL)	0.04(0.04-0.05)	0.06(0.04-0.07)	0.09(0.06-0.13)	**<.0001**
		N=460	N=478	N=254	
	Benzene (ng/mL)	0.02(0.02-0.02)	0.03(0.02-0.03)	0.03(0.02-0.04)	0.0599
		N=463	N=476	N=260	
	Toluene (ng/mL)	0.07(0.06-0.08)	0.09(0.08-0.10)	0.09(0.08-0.11)	**0.0022**
		N=451	N=452	N=250	
	Methyl-tert-butyl ether (MTBE) (ng/mL)	0.007(0.007-0.007)	0.007(0.007-0.007)	0.007(0.007-0.007)	0.8471

Cotinine		N=953	N=1,011	N=512	
	Cotinine (ng/mL)	0.08(0.06-0.10)	0.28(0.19-0.41)	0.59(0.34-1.01)	**<.0001**
Metals		N=340	N=353	N=187	
	Arsenic, total (ug/L)	6.05(5.12-7.15)	5.39(4.51-6.43)	5.77(4.68-7.10)	0.5497
	Cadmium (ug/L)*	0.10(0.09-0.12)	0.11(0.09-0.14)	0.15(0.13-0.18)	**0.0421**
		N=492	N=501	N=270	
	Lead (ug/dL)	0.60(0.54-0.66)	0.56(0.52-0.60)	0.59(0.54-0.65)	0.5573
	Mercury, total (ug/L)*	0.83(0.72-0.96)	0.63(0.57-0.70)	0.58(0.51-0.66)	**<.0001**
Pesticides		N=304	N=342	N=160	
	3-(Ethlycarbamoyl) benzoic acid (DEET acid) (ng/mL)	2.21(1.45-3.37)	2.76(2.04-3.75)	2.79(2.01-3.87)	0.3480
		N=290	N=349	N=159	
	Hexachlorobenzene (HCB) (pg/g)	41.11(39.50-42.78)	41.22(39.69-42.80)	43.86(41.53-46.33)	0.0602
	Oxychlordane (OXYCHLOR) (pg/g)*	22.80(21.07-24.67	20.81(19.13-22.63)	22.56(20.02-25.42)	0.0677
Environmental Phenols		N=289	N=344	N=156	
	Bisphenol A (ng/mL)	0.95(0.68-1.31)	1.15(0.82-1.61)	1.29(1.09-1.53)	**0.0045**
Per- and polyfluoroalkyl substances (PFAs)		N=275	N=325	N=152	
	n-perfluorooctanoic acid (n-PFOA) (ng/mL)	1.23(1.09-1.38)	1.07(0.95-1.20)	0.99(0.89-1.09)	**<.0001**
	n-perfluorooctane sulfonic acid (n-PFOS) (ng/mL)*	2.27(2.02-ag2.56)	2.22(1.94-2.53)	1.74(1.51-2.00)	**0.0026**
		N=288	N=349	N=158	
	Perfluorohexane sulfonic acid (PFHxS) (ng/mL)	0.66(0.53-0.81)	0.66(0.53-0.83)	0.53(0.43-0.66)	**0.0008**
	Perfluorononanoic acid (PFNA) (ng/mL)*	0.41(0.35-0.47)	0.42(0.34-0.50)	0.36(0.29-0.45)	**0.0115**

[1]*p*-values were obtained using Kruskal-Wallis Test.

[2]Boldface indicates p-value <0.05.

*Chemicals without n's have the same n's as the chemical listed directly above.

Table S8. Environmental chemical distributions across body mass index levels.

**Environmental Classes**	**Environmental Chemicals**	**Underweight**	**Normal**	**Overweight**	**Obese**	***p*-value**
		**Median (Range)**	**Median (Range)**	**Median (Range)**	**Median (Range)**	
Brominated Flame Retardants (BFRs)		N=31	N=276	N=193	N=298	
	2,2',4,4',5,5'-Hexabromobiphenyl (PBB-153) (pg/g)	3.44(2.67-4.43)	5.55(4.59-6.71)	5.53(4.75-6.44)	5.28(4.42-6.32)	**0.0057**
	2,4,4'-Tribromodiphenyl ether (PBDE-28) (pg/g)*	3.60(3.09-4.19)	3.96(3.67-4.27)	4.07(3.71-4.48)	4.04(3.77-4.32)	0.5742

	2,2',4,4'-Tetrabromodiphenyl ether (PBDE-47) (pg/g)*	69.30(60.56-79.29)	74.49(68.99-80.43)	78.58(70.11-88.08)	76.29(71.19-81.75)	0.5409
	2,2',4,4',5-Pentabromodiphnyl ether (PBDE-99) (pg/g)*	13.40(11.50-15.60)	14.05(12.79-15.44)	15.33(13.27-17.70)	14.50(13.43-15.65)	0.3524
	2,2',4,4',6-Pentabromodiphyl ether (PBDE-100) (pg/g)*	14.72(13.73-15.78)	15.98(14.63-17.46)	17.13(15.32-19.17)	16.66(15.49-17.91)	0.0945
	2,2',4,4',5,5'-Hxbromodiphnyl ether (PBDE-153) (pg/g)*	41.18(39.62-42.80)	50.42(46.51-54.66)	46.36(41.81-51.42)	43.28(39.63-47.26)	0.2856
Volatile Organic Compounds (VOCs)		N=40	N=407	N=287	N=480	
	1,4-Dichlorobenzene (ng/mL)	0.09(0.05-0.16)	0.04(0.04-0.05)	0.06(0.04-0.07)	0.06(0.05-0.08)	0.2107
		N=39	N=392	N=281	N=480	
	Benzene (ng/mL)	0.03(0.02-0.05)	0.02(0.02-0.03)	0.03(0.02-0.03)	0.03(0.02-0.03)	0.1466
		N=40	N=399	N=279	N=481	
	Toluene (ng/mL)	0.07(0.04-0.13)	0.08(0.07-0.09)	0.08(0.06-0.10)	0.08(0.07-0.10)	0.5159
		N=38	N=393	N=269	N=453	
	Methyl-tert-butyl ether (MTBE) (ng/mL)	0.007(0.007-0.007)	0.007(0.007-0.007)	0.007(0.007-0.007)	0.007(0.007-0.007)	0.2687
Cotinine		N=88	N=846	N=592	N=950	
	Cotinine (ng/mL)	0.44(0.14-1.35)	0.13(0.09-0.17)	0.19(0.13-0.29)	0.22(0.15-0.32)	**0.0070**
Metals		N=28	N=299	N=204	N=349	
	Arsenic, total (ug/L)	5.38(3.88-7.46)	5.58(4.64-6.71)	5.80(4.81-6.98)	5.88(5.06-6.84)	0.8714
	Cadmium (ug/L)*	0.10(0.07-0.15)	0.09(0.07-0.11)	0.64(0.54-0.76)	0.15(0.13-0.18)	**0.0007**
		N=42	N=424	N=295	N=502	
	Lead (ug/dL)	0.60(0.47-0.77)	0.61(0.55-0.68)	0.63(0.56-0.72)	0.53(0.49-0.56)	**0.0310**
	Mercury, total (ug/L)*	0.58(0.49-0.67)	0.87(0.74-1.01)	0.71(0.60-0.83)	0.59(0.53-0.65)	**<.0001**
Pesticides		N=27	N=284	N=189	N=306	
	3-(Ethlycarbamoyl) benzoic acid (DEET acid) (ng/mL)	1.97(1.25-3.10)	1.84(1.26-2.69)	3.10(2.22-4.31)	3.13(2.34-4.18)	**0.0057**
		N=31	N=276	N=193	N=298	
	Hexachlorobenzene (HCB) (pg/g)	38.87(34.26-44.11)	41.96(40.40-43.57)	41.98(39.85-44.22)	41.09(39.31-42.96)	0.3329
	Oxychlordane (OXYCHLOR) (pg/g)*	14.60(12.01-17.74)	22.93(21.19-24.82)	22.14(19.62-24.98)	21.45(19.54-23.55)	**0.0024**

Environmental Phenols		N=30	N=271	N=192	N=296	
	Bisphenol A (ng/mL)	0.95	0.88(0.63-1.22)	1.00(0.68-1.47)	1.40(1.04-1.88)	**0.0005**
Per- and polyfluoroalkyl substances (PFAs)		N=25	N=264	N=181	N=282	
	n-perfluorooctanoic acid (n-PFOA) (ng/mL)	1.33(0.43-4.12)	1.21(1.07-1.36)	1.22(0.97-1.55)	0.95(0.84-1.08)	**<.0001**
	n-perfluorooctane sulfonic acid (n-PFOS) (ng/mL)*	2.17(0.06-68.91)	2.27(2.00-2.58)	2.35(2.02-2.73)	1.91(1.74-2.11)	**0.0013**
		N=29	N=275	N=193	N=298	
	Perfluorohexane sulfonic acid (PFHxS) (ng/mL)	1.19	0.58(0.47-0.71)	0.76(0.59-0.98)	0.60(0.49-0.74)	**0.0088**
	Perfluorononanoic acid (PFNA) (ng/mL)*	0.53	0.42(0.36-0.48)	0.43(0.35-0.53)	0.36(0.31-0.43)	**<.0001**

[1]*p*-values were obtained using Kruskal-Wallis Test.

[2]Boldface indicates p-value <0.05.

[*]Confidence intervals that were not provided were due to the small sample size.

Table S9. Environmental chemical distribution across smoking status.

Environmental Classes	Environmental Chemicals	Never Smoker	Former Smoker	Current Smoker	*p*-value
		**Median (Range)**	**Median (Range)**	**Median (Range)**	
Brominated Flame Retardants (BFRs)		N=592	N=75	N=131	
	2,2',4,4',5,5'-Hexabromobiphenyl (PBB-153) (pg/g)	5.09(4.34-5.97)	6.27(4.89-8.05)	6.03(4.86-7.48)	**0.0015**
	2,4,4'-Tribromodiphenyl ether (PBDE-28) (pg/g)*	3.96(3.79-4.14)	4.14(3.57-4.81)	4.09(3.74-4.47)	0.2000
	2,2',4,4'-Tetrabromodiphenyl ether (PBDE-47) (pg/g)*	75.26(72.10-78.55)	81.81(71.44-93.70)	74.96(67.98-82.67)	0.1983
	2,2',4,4',5-Pentabromodiphnyl ether (PBDE-99) (pg/g)*	14.34(13.55-15.18)	15.82(13.33-18.78)	14.24(12.59-16.10)	0.1851
	2,2',4,4',6-Pentabromodiphyl ether (PBDE-100) (pg/g)*	16.38(15.51-17.30)	17.30(14.99-19.97)	16.18(14.72-17.77)	0.2773
	2,2',4,4',5,5'-Hxbromodiphnyl ether (PBDE-153) (pg/g)*	44.94(41.72-48.40)	49.63(43.86-56.17)	51.60(45.52-58.48)	**0.0021**
Volatile Organic Compounds (VOCs)		N=891	N=127	N=196	
	1,4-Dichlorobenzene (ng/mL)	0.06(0.05-0.07)	0.04(0.03-0.06)	0.05(0.04-0.06)	**<.0001**
		N=876	N=124	N=192	
	Benzene (ng/mL)	0.02(0.01-0.02)	0.03(0.02-0.03)	0.11(0.09-0.14)	**<.0001**
		N=881	N=125	N=193	
	Toluene (ng/mL)	0.06(0.05-0.06)	0.08(0.06-0.09)	0.30(0.25-0.36)	**<.0001**
		N=844	N=121	N=181	
	Methyl-tert-butyl ether (MTBE) (ng/mL)	0.007(0.007-0.007)	0.007(0.007-0.007)	0.007(0.007-0.007)	0.8831
Cotinine		N=1,812	N=255	N=409	
	Cotinine (ng/mL)	0.03(0.03-0.04)	0.13(0.08-0.21)	89.58(71.78-111.78)	**<.0001**
Metals		N=639	N=93	N=148	
	Arsenic, total (ug/L)	5.53(4.82-6.34)	5.98(4.81-7.42)	6.39(5.15-7.91)	0.6700
	Cadmium (ug/L)*	0.09(0.08-0.11)	0.15(0.12-0.19)	0.18(0.15-0.21)	**<.0001**
		N=924	N=132	N=207	
	Lead (ug/dL)	0.53(0.49-0.57)	0.67(0.60-0.74)	0.76(0.71-0.82)	**<.0001**

	Mercury, total (ug/L)*	0.68(0.62-0.74)	0.97(0.83-1.13)	0.63(0.54-0.74)	**0.0008**
Pesticides		N=596	N=75	N=135	
	3-(Ethlycarbamoyl) benzoic acid (DEET acid) (ng/mL)	2.20(1.71-2.83)	2.89(1.86-4.48)	3.72(2.07-6.67)	**0.0065**
		N=592	N=75	N=131	
	Hexachlorobenzene (HCB) (pg/g)	41.26(39.91-42.65)	42.46(40.72-44.28)	42.23(40.29-44.25)	0.1326
	Oxychlordane (OXYCHLOR) (pg/g)*	20.81(19.28-22.45)	24.23(21.80-26.94)	25.33(22.77-28.18)	**0.0007**
Environmental Phenols		N=589	N=73	N=127	
	Bisphenol A (ng/mL)	1.08(0.87-1.33)	0.92(0.58-1.47)	1.16(0.76-1.78)	**0.0078**
Per- and polyfluoroalkyl substances (PFAs)		N=554	N=70	N=128	
	n-perfluorooctanoic acid (n-PFOA) (ng/mL)	1.05(0.95-1.17)	1.29(1.04-1.60)	1.29(1.01-1.65)	0.0827
	n-perfluorooctane sulfonic acid (n-PFOS) (ng/mL)*	2.24(2.03-2.46)	2.09(1.79-2.43)	1.92(1.65-2.24)	0.0818
		N=589	N=75	N=131	
	Perfluorohexane sulfonic acid (PFHxS) (ng/mL)	0.60(0.51-0.70)	0.81(0.62-1.08)	0.71(0.54-0.95)	0.0606
	Perfluorononanoic acid (PFNA) (ng/mL)*	0.39(0.34-0.43)	0.53(0.34-0.83)	0.40(0.34-0.48)	0.3934

[1]*p*-values were obtained using Kruskal-Wallis Test.

[2]Boldface indicates p-value <0.05.

*Confidence intervals that were not provided were due to the small sample size.

Table S10. Environmental chemical distribution across alcohol use.

**Environmental Classes**	**Environmental Chemicals**	**Yes**	**No**	***p*-value**
		**Median (Range)**	**Median (Range)**	
Brominated Flame Retardants (BFRs)		N=509	N=289	
	2,2',4,4',5,5'-Hexabromobiphenyl (PBB-153) (pg/g)	5.71(4.83-6.75)	4.59(3.95-5.33)	**0.0449**
	2,4,4'-Tribromodiphenyl ether (PBDE-28) (pg/g)*	3.93(3.72-4.16)	4.19(3.90-4.50)	0.1677
	2,2',4,4'-Tetrabromodiphenyl ether (PBDE-47) (pg/g)*	73.94(69.90-78.20)	81.29(75.27-87.78)	0.0863
	2,2',4,4',5-Pentabromodiphnyl ether (PBDE-99) (pg/g)*	13.93(13.07-14.85)	16.00(14.53-17.63)	**0.0237**
	2,2',4,4',6-Pentabromodiphyl ether (PBDE-100) (pg/g)*	15.84(14.92-16.81)	18.13(16.54-19.87)	**0.0301**

	2,2',4,4',5,5'-Hxbromodiphnyl ether (PBDE-153) (pg/g)*	48.95(45.54-52.61)	40.97(37.81-44.39)	**0.0032**
Volatile Organic Compounds (VOCs)		N=756	N=458	
	1,4-Dichlorobenzene (ng/mL)	0.05(0.04-0.06)	0.08(0.06-0.10)	**<.0001**
		N=740	N=452	
	Benzene (ng/mL)	0.03(0.02-0.03)	0.02(0.02-0.02)	**<.0001**
		N=744	N=455	
	Toluene (ng/mL)	0.09(0.08-0.10)	0.06(0.05-0.08)	**<.0001**
		N=714	N=439	
	Methyl-tert-butyl ether (MTBE) (ng/mL)	0.007(0.007-0.007)	0.007(0.007-0.007)	0.6128
Cotinine		N=1,542	N=934	
	Cotinine (ng/mL)	0.28(0.22-0.35)	0.06(0.04-0.09)	**<.0001**
Metals		N=538	N=342	
	Arsenic, total (ug/L)	5.82(5.08-6.66)	5.57(4.84-6.40)	0.5620
	Cadmium (ug/L)*	0.11(0.10-0.13)	0.12(0.10-0.14)	0.7418
		N=787	N=476	
	Lead (ug/dL)	0.61(0.57-0.65)	0.53(0.48-0.58)	**0.0051**
	Mercury, total (ug/L)*	0.76(0.70-0.82)	0.58(0.50-0.69)	**<.0001**
Pesticides		N=504	N=302	
	3-(Ethlycarbamoyl) benzoic acid (DEET acid) (ng/mL)	2.71(1.90-3.86)	2.04(1.59-2.61)	**0.0442**
		N=509	N=289	
	Hexachlorobenzene (HCB) (pg/g)	41.90(40.45-43.41)	40.71(38.55-42.99)	0.6944
	Oxychlordane (OXYCHLOR) (pg/g)*	22.99(21.41-24.69)	19.40(17.50-21.49)	**0.0259**
Environmental Phenols		N=502	N=289	
	Bisphenol A (ng/mL)	1.13(0.84-1.52)	0.91(0.78-1.07)	**0.0224**
Per- and polyfluoroalkyl substances (PFAs)		N=481	N=271	
	n-perfluorooctanoic acid (n-PFOA) (ng/mL)	1.22(1.08-1.37)	0.89(0.77-1.04)	**<.0001**

	n-perfluorooctane sulfonic acid (n-PFOS) (ng/mL)*	2.17(1.98-2.38)	2.12(1.79-2.50)	0.3104
		N=506	M=289	
	Perfluorohexane sulfonic acid (PFHxS) (ng/mL)	0.71(0.60-0.85)	0.47(0.39-0.55)	**<.0001**
	Perfluorononanoic acid (PFNA) (ng/mL)*	0.41(0.36-0.46)	0.39(0.32-0.48)	**0.0408**

[1]*p*-values were obtained using Kruskal-Wallis Test.

[2]Boldface indicates p-value <0.05.

*Confidence intervals that were not provided were due to the small sample size.

Table S11. Environmental chemical distribution across infertility status.

**Environmental Classes**	**Environmental Chemicals**	**Infertile**	**Fertile**	***p*-value**
		**Median (Range)**	**Median (Range)**	
Brominated Flame Retardants (BFRs)		N=89	N=709	
	2,2',4,4',5,5'-Hexabromobiphenyl (PBB-153) (pg/g)	7.74(5.70-10.50)	5.06(4.47-5.73)	**<.0001**
	2,4,4'-Tribromodiphenyl ether (PBDE-28) (pg/g)*	4.21(3.73-4.74)	3.97(3.79-4.15)	0.2364
	2,2',4,4'-Tetrabromodiphenyl ether (PBDE-47) (pg/g)*	77.73(69.51-86.93)	75.62(71.97-79.44)	0.5494
	2,2',4,4',5-Pentabromodiphnyl ether (PBDE-99) (pg/g)*	14.35(12.57-16.37)	14.50(13.72-15.33)	0.7926
	2,2',4,4',6-Pentabromodiphyl ether (PBDE-100) (pg/g)*	16.89(14.73-19.38)	16.37(15.43-17.37)	0.6423
	2,2',4,4',5,5'-Hxbromodiphnyl ether (PBDE-153) (pg/g)*	50.92(44.89-57.76)	45.92(43.09-48.94)	**0.0415**
Volatile Organic Compounds (VOCs)		N=141	N=1,073	
	1,4-Dichlorobenzene (ng/mL)	0.06(0.04-0.08)	0.05(0.04-0.06)	0.0762
		N=139	N=1,053	
	Benzene (ng/mL)	0.02(0.02-0.03)	0.02(0.02-0.03)	0.5734
		N=141	N=1,058	
	Toluene (ng/mL)	0.08(0.06-0.09)	0.08(0.07-0.09)	0.8239
		N=131	N=1,022	
	Methyl-tert-butyl ether (MTBE) (ng/mL)	0.007(0.006-0.007)	0.007(0.007-0.007)	0.6957
Cotinine		N=284	N=2,192	
	Cotinine (ng/mL)	0.17(0.09-0.30)	0.18(0.14-0.22)	0.6168
Metals		N=105	N=775	
	Arsenic, total (ug/L)	6.67(5.10-8.73)	5.62(5.04-6.26)	0.0934
	Cadmium (ug/L)*	0.14(0.11-0.18)	0.11(0.10-0.12)	**0.0276**
		N=151	N=1,112	

	Lead (ug/dL)	0.62(0.57-0.67)	0.58(0.54-0.61)	0.1644
	Mercury, total (ug/L)*	0.73(0.61-0.88)	0.70(0.65-0.75)	0.2457
Pesticides		N=95	N=711	
	3-(Ethlycarbamoyl) benzoic acid (DEET acid) (ng/mL)	2.62(1.58-4.35)	2.49(1.88-3.29)	0.9908
		N=89	N=709	
	Hexachlorobenzene (HCB) (pg/g)	44.62(41.90-47.52)	41.09(39.83-42.39)	**0.0031**
	Oxychlordane (OXYCHLOR) (pg/g)*	26.54(23.65-29.79)	21.26(19.90-22.71)	**<.0001**
Environmental Phenols		N=87	N=702	
	Bisphenol A (ng/mL)	1.09(0.57-2.07)	1.07(0.84-1.36)	0.5277
Per- and polyfluoroalkyl substances (PFAs)		N=86	N=666	
	n-perfluorooctanoic acid (n-PFOA) (ng/mL)	0.86(0.69-1.06)	1.17(1.06-1.30)	0.0618
	n-perfluorooctane sulfonic acid (n-PFOS) (ng/mL)*	1.78(1.49-2.12)	2.23(2.07-2.40)	0.0886
		N=88	N=707	
	Perfluorohexane sulfonic acid (PFHxS) (ng/mL)	0.68(0.43-1.07)	0.63(0.54-0.74)	0.1159
	Perfluorononanoic acid (PFNA) (ng/mL)*	0.32(0.24-0.42)	0.42(0.37-0.47)	0.1286

[1]*p*-values were obtained using Kruskal-Wallis Test.

[2]Boldface indicates p-value <0.05.

*Confidence intervals that were not provided were due to the small sample size.

Table S12. Logistic regression analysis estimating the associations between exposure score and infertility.

		**50th Percentile[a]**				**75th Percentile[a]**	
**Independent Variable**		**Odds Ratio (95% CI)**	**Odds Ratio (95% CI)**	**Independent Variable**		**Odds Ratio (95% CI)**	**Odds Ratio (95% CI)**
	**N**	**Unadjusted Model**	**Adjusted Model[b]**		**N**	**Unadjusted Model**	**Adjusted Model[b]**
**Exposure score continuous (0-18)**	2579	1.02(0.97-1.06)	1.01(0.96-1.06)	**Exposure score continuous (0-17)**	2535	1.02(0.97-1.07)	0.99(0.94-1.06)
**Exposure score categories**	**N**			**Exposure score categories[a]**	**N**		
Low exposure (0-1)	761	[ref.]	[ref.]	Low exposure (0-1)	799	[ref.]	[ref.]
Moderate low exposure (2-3)	582	1.16(0.79-1.71)	1.22(0.83-1.79)	Moderate low exposure (2-3)	592	0.95(0.60-1.50)	1.04(0.66-1.63)

Moderate high exposure (4-6)	696	1.35(0.88-2.08)	1.51(0.97-2.33)	Moderate high exposure (4-6)	635	1.40(0.96-2.04)	1.53(1.00-2.31)
High exposure (7-18)	540	1.29(0.83-2.03)	1.22(0.74-2.01)	High exposure (7-17)	553	1.24(0.77-2.00)	1.15(0.69-1.91)

[a]Exposure score categories are based on quartiles.

[b]Adjusted model controlled for age, race, annual family income, education, and smoking status.
